# Linkage disequilibrium and signatures of selection on chromosomes 19 and 29 in beef and dairy cattle

**DOI:** 10.1111/j.1365-2052.2008.01772.x

**Published:** 2008-12

**Authors:** A Prasad, R D Schnabel, S D McKay, B Murdoch, P Stothard, D Kolbehdari, Z Wang, J F Taylor, S S Moore

**Affiliations:** * Department of Agricultural, Food and Nutritional Science, University of AlbertaEdmonton, Alberta, Canada T6G 2P5; † Division of Animal Sciences, University of MissouriColumbia, MO 65211, USA

**Keywords:** bovine, linkage disequilibrium, selection, single nucleotide polymorphism

## Abstract

The objective of this study was to quantify the extent of linkage disequilibrium (LD) on bovine chromosomes 19 and 29 and to study the pattern of selection signatures in beef and dairy breeds (Angus and Holstein) of *Bos taurus*. The extent of LD was estimated for 370 and 186 single nucleotide polymorphism markers on BTA19 and 29 respectively using the square of the correlation coefficient (*r*^2^) among alleles at pairs of loci. A comparison of the extent of LD found that the decline of LD followed a similar pattern in both breeds. We observed long-range LD and found that LD dissipates to background levels at a locus separation of about 20 Mb on both chromosomes. Along each chromosome, patterns of LD were variable in both breeds. We find that a minimum of 30 000 informative and evenly spaced markers would be required for whole-genome association studies in cattle. In addition, we have identified chromosomal regions that show some evidence of selection for economically important traits in Angus and Holstein cattle. The results of this study are of importance for the design and application of association studies.

## Introduction

Linkage disequilibrium (LD) is the non-random association of alleles at different loci. If two alleles at two different loci are in LD, combinations of alleles within haplotypes occur at frequencies that differ from that expected under the hypothesis of independence. An association between the genetic variation at a locus and a phenotype indicates that either the genetic variation at that locus directly affects the phenotype of interest or the locus is in LD with the causal mutation (Mueller 2004). The feasibility of association studies depend strongly on the extent of LD, which determines how many markers should be typed in a genome scan to detect a quantitative trait locus (QTL) using LD.

The first whole-genome LD study in cattle, to quantify the extent and pattern of LD, was performed using 284 microsatellite markers sampled from 581 maternally inherited gametes in Dutch black and white dairy cattle, where high levels of LD extended over several tens of centimorgans (Farnir *et al.* 2000). Several subsequent studies have confirmed extensive LD in cattle (Tenesa *et al.* 2003; Vallejo *et al.* 2003; Khatkar *et al.* 2006a; Odani *et al.* 2006). Only recently, a study performed in a large mildly selected cattle population from Western Africa under an extensive breeding system has shown that LD extends over shorter distances than the previous studies from developed countries, which was explained by increasing selective pressure and/or by an admixture process (Thevenon *et al.* 2007). All these LD studies were performed using very informative microsatellite loci, but at a relatively low locus density. However, with the completion of the bovine genome sequencing project, it has become possible to estimate the extent of LD using dense single nucleotide polymorphism (SNP) marker maps, thereby dramatically increasing resolution. In addition to their abundance in the genome (Snelling *et al.* 2005), SNP markers have low genotyping costs (Hinds *et al.* 2005). Khatkar *et al.* (2006b) reported a first-generation LD map of bovine chromosome 6 in Australian Holstein–Friesian cattle using SNP loci and estimated the extent of LD using *D*′. The distance over which LD is likely to be useful for association mapping was found to be 13.3 Mb confirming that the range of LD is extensive in Holstein–Friesian dairy cattle. McKay *et al.* (2007) generated LD maps for eight breeds of cattle from the *Bos taurus* and *Bos indicus* subspecies using 2670 SNP markers and observed that the extent of LD (estimated using *r*^2^) available for association analysis does not exceed 500 kb. The differences in the extent of LD between the study by McKay *et al.* (2007) and previous studies were attributed to the differences in measures used to report LD, which is specifically *D*′ vs. *r*^2^. *D*′ has been reported to overestimate the extent of LD (Ardlie *et al.* 2002; Ke *et al.* 2004) thus resulting in extensive LD at long intermarker distances in previous studies (Farnir *et al.* 2000; Tenesa *et al.* 2003; Vallejo *et al.* 2003; Khatkar *et al.* 2006a; Odani *et al.* 2006).

Here, we report a study of the extent of LD on chromosomes 19 and 29 and of the pattern of selection signatures on these chromosomes in *Bos taurus* beef and dairy breeds (Angus and Holstein) using dense SNP markers. We have chosen BTA19 and BTA29 as candidate chromosomes for mapping because QTL for several economically important traits has been identified on these chromosomes (Stone *et al.* 1999; Mosig *et al.* 2001; MacNeil & Grosz 2002; Casas *et al.* 2003; Li *et al.* 2004; Ashwell *et al.* 2005; Nkrumah *et al.* 2007). The information generated from this study, with a relatively large number of animals per breed compared to other studies, has important implications for the design and application of association studies in cattle populations as well as for selective breeding programmes.

## Materials and methods

### Collection of DNA samples

DNA was collected from Angus (*n*=126, USA) and Holstein (*n*=321, Semex Alliance) cattle. To maximize the genetic diversity within each sampled population, families were selected to span the diversity of each breed. Three-generation families were sampled so that chromosomes could be phased using linkage information. The general family structure consisted of a grandparent, parent and three or more progeny.

### Marker selection and genotyping

A total of 1001 and 535 evenly spaced SNP markers for BTA19 and 29 respectively were chosen from bovine sequence build 2.0 (ftp://ftp.hgsc.bcm.tmc.edu/pub/data/Btaurus/snp). The markers were genotyped within each population of beef and dairy animals using the Illumina BeadStation 500G genotyping system (Oliphant *et al.* 2002). However, only 555 and 253 SNP markers from BTA19 and 29 respectively were used for the LD analysis. These loci had successfully been mapped on the high-resolution 12 000 rad radiation hybrid panel and were considered to be correctly ordered on both BTA19 and 29 (Prasad *et al.* 2007). Some loci did not amplify in the genotyped animals and those loci that were monomorphic or that had a minor allele frequency (MAF) < 0.03 were removed from the study. After these filtering procedures, the LD analysis was performed using 370 and 367 markers on BTA19 and 186 and 179 markers on BTA29 for the Angus and Holstein populations respectively. The sequence and the NCBI IDs of the SNPs used in the LD analysis are described in the work of Prasad *et al.* (2007). To test whether Holstein differ significantly from Angus in the distribution of MAF, the proc freq procedure in sas(v. 9.1, SAS, Inc.) was run using a two-way contingency table of loci against breeds.

### Marker positions

Genomic sequence coordinates for SNPs were obtained by performing blast (Altschul *et al.* 1990) comparisons between SNP-flanking sequences and the 7.1× bovine genome assembly (Btau 3.1). The marker order and their corresponding genomic coordinates were corrected if they disagreed with the RH map order of Prasad *et al.* (2007). For each chromosome, a bp/cR conversion ratio was estimated by dividing the highest base-pair position by its corresponding cR position. The resultant ratios were 13 816.49 and 15 413.2 for BTA19 and 29 respectively. The relative positions of markers (in bp) were estimated by multiplying the conversion ratio with the RH position. Markers that could not be separated by their RH positions were ordered according to their order in the bovine genome sequence assembly; RH mapping has difficulty ordering closely linked markers, although the sequence assembly is accurate at a fine scale. The list of SNPs used in the LD analyses and their inferred chromosomal positions in base pairs are in Table S1.

### Estimation of phased haplotypes

We used genoprob v2.0(Thallman *et al.* 2001a,b) for data-quality checking and estimated phased haplotypes based on the pedigree and estimated recombination rates, which were set proportional to the physical distances among the loci. Both the pedigree and the marker locations (map) were used to estimate the segregation of alleles throughout the entire pedigree. By tracing closely linked markers through a multigenerational pedigree, the linkage phase of the alleles was inferred. A set of five loci on BTA29 were chosen to illustrate this point, as shown in Table S2. Four progeny of sire 2672891 inherited alternate haplotypes from this sire (blue/yellow in Table S2). Sire 2672891 inherited the yellow haplotype from his maternal granddam. This represents only a small proportion of the markers on this chromosome; there were 22 markers centromeric of the first marker shown and an additional ∼150 markers telomeric of this region. The combination of all pedigree and map information available allowed the accurate reconstruction of whole-chromosome-length haplotypes via linkage. genoprob estimates the probability that a genotype is correct (pGmx) and the order (phase) of the allele is correct (oGmx) conditional on the pedigree, locus order and map distances. For the LD analysis, we excluded all genotypes with pGmx ≤ 0.95, but did not put any constraint on oGmx. The summary of average genotype and order probabilities for each breed is shown in Table 1. As evident from Table 1, more than 90% of the genotypes have order (phase) probabilities >0.95.

**Table 1:** Summary of the proportion of genotypes in different ranges of probabilities that a genotype is correct (pGmx) and that the order or the phase of the allele is correct (oGmx) for Angus and Holstein.

	pGmx		oGmx	
Range	Holstein	Angus	Holstein	Angus
<0.9000	0.0072	0.0174	0.0107	0.1140
0.9000–0.9500	0.0019	0.0027	0.0067	0.0279
0.9500–0.9900	0.0239	0.0104	0.0176	0.0567
0.9900–0.9990	0.3992	0.6831	0.0334	0.1679
≥0.999	0.5679	0.2864	0.8994	0.6336

### Estimation of linkage disequilibrium

Linkage disequilibrium was measured as the square of the correlation coefficient (*r*^2^) between marker alleles using gold (Hill & Robertson 1968; Abecasis & Cookson 2000). Only maternally inherited haplotypes were used to estimate LD in this study to avoid the over-representation of paternal haplotypes within the essentially all-male pedigrees. The *r*^2^-values for all pairwise combinations of markers were binned according to the physical distances separating the markers. The average number of locus pairs within each intermarker distance bin for both breeds are shown in Table 2. The graphical representation of the patterns of LD along the chromosomes was generated using the gold package.

**Table 2:** Total number of locus pairs by intermarker distances in Angus and Holstein averaged over BTA19 and 29.

Intermarker distances	Holstein	Angus
5 kb	25	29.5
50 kb	71.5	80.5
100 kb	90.5	91.5
250 kb	232.5	244
500 kb	408.5	447.5
1 Mb	794.5	835.5
2 Mb	1473	1530
5 Mb	3933	4038.5
7 Mb	2463	2425.5
10 Mb	3424	3482.5
20 Mb	10160	10340
40 Mb	12880	13171
65 Mb	5590.5	6019

### Estimation of signatures of selection

We computed allelic frequencies for those SNPs whose genotypes were scored in both breeds. There were 334 and 165 such markers on BTA19 and 29 respectively that were used for the LD study. For estimating signatures of selection, we also included markers that were fixed in one breed but that were still segregating in the other breed. There were additionally 21 and 10 such markers on BTA19 and BTA29 respectively that were included in this analysis. However, these markers had been excluded from the LD study because their MAF values were <0.03. Therefore, in total, the estimation of signatures of selection was carried out using 355 and 175 markers on BTA19 and BTA29 respectively. We also computed rolling average allele frequencies in both breeds (using the frequency of the allele with the lowest frequency averaged over both breeds) using a five-locus sliding window for both chromosomes and for each pair of averages; we subtracted the mean Angus allele frequency from that for Holstein. We plotted mean allele frequency differences against the location of the third locus within the five-locus window. To establish whether the allele frequency difference between the breeds differed significantly from zero and thus was putatively indicative of a selection signature, we performed 100 000 and 1 000 000 allele-frequency-against-locus permutation tests for BTA19 and BTA29 respectively to empirically identify the 5% significance level thresholds. To confirm the chromosomal regions identified using the sliding-window approach, we performed a chromosome-wide scan to detect regions showing evidence of selection using a Web-based tool to compute the extended haplotype homozygosity (EHH) statistic (Mueller & Andreoli 2004). First, the haplotypes at the locus of interest (core haplotype) were identified and the decay of LD as a function of increasing distance from the core haplotype as measured by EHH was evaluated (Sabeti *et al.* 2002). The test for positive selection requires identification of a core haplotype with a combination of high frequency and high EHH, as compared to other core haplotypes at the locus (Sabeti *et al.* 2002). Again, only maternally inherited haplotypes were used for this analysis.

## Results and discussion

**Figure 1:**
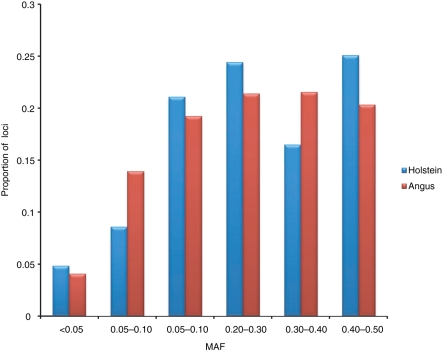
The distribution of minor allele frequencies for the SNP markers (MAF > 0.03) used in the LD analysis on BTA19 and BTA29.

The average MAF for SNPs on BTA19 was 0.27 for both Angus and Holstein, but was 0.25 and 0.27, respectively on BTA29. The distribution of MAFs for SNPs used in the LD analyses in both breeds is shown in Fig. 1. It is evident from this figure that MAF distribution deviates from uniform and Holstein differs from Angus (*P*<0.001) for its MAF distribution. The presence of a uniform distribution of SNP MAFs is because of the ascertainment bias in SNP discovery and does not represent the true distribution of SNP MAF in the genome, which is more appropriately modelled by a gamma distribution. Any difference between the MAF distributions probably reflects a breed of ascertainment effect (i.e. the SNPs were discovered because they were the most common SNPs on these chromosomes in Holstein), which would lead to an excess of high MAF SNPs in one breed and an excess of low MAF in the second. Therefore, the difference probably has no biological significance other than identifying the breed of SNP discovery. The distribution of MAF for SNPs used in the estimation of selection signatures using the five-locus sliding-window approach (results not shown) was not different from Fig. 1, showing that the focus on these SNPs did not introduce an ascertainment bias.

**Figure 2:**
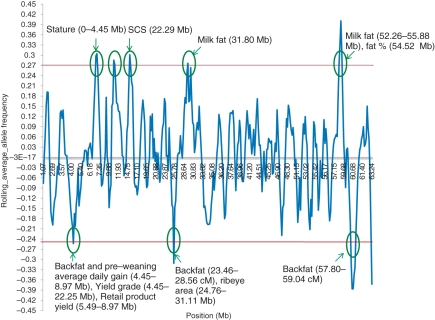
Rolling average allele frequency distribution of 355 SNP markers along BTA19 for beef and dairy cattle. The deviations above and below the axis show evidence of selection in dairy and beef cattle respectively with significant thresholds of 0.27 and −0.25 respectively shown by red lines.

**Figure 3:**
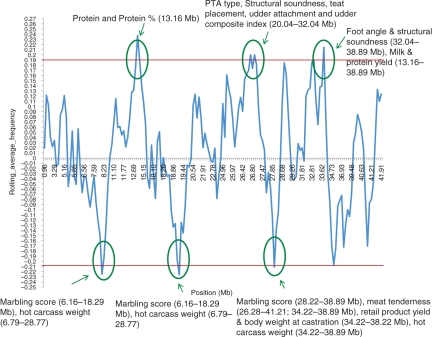
Rolling average allele frequency distribution of 175 SNP markers along BTA29 for beef and dairy cattle. The deviations above and below the axis show evidence of selection in dairy and beef cattle with significant thresholds of 0.19 and −0.21 respectively shown by red lines.

Details of the allelic frequencies for both breeds are provided in Table S3. After plotting the differences in rolling average allele frequencies between beef and dairy cattle against the third locus coordinate within a five-locus sliding window, we observed large fluctuations about the axis on both chromosomes (Figs 2 & 3). The allele frequency thresholds required to achieve statistical significance were found by performing permutation tests and were 0.27 and −0.25 on BTA19 and 0.19 and −0.21 on BTA29 respectively (shown by the red-coloured lines in Figs 2 & 3). In total, we tested 351 and 171 sliding windows for BTA19 and 29 respectively and the number of chromosomal regions identified because differing between the breeds was greater than expected by chance. We found evidence of selection in five regions (6.18–7.35, 9.88–11.93, 14.75–17.10, 28.64–30.83 and 57.15–59.68 Mb) in Holstein and three regions (4.00–5.40, 24–26 and 60–61 Mb) in Angus on BTA19. On BTA29, there were three regions (11.77–15.15, 26.42–27.47 and 33–34 Mb) in Holstein and three regions (7.5–8.50, 18.75–19.45 and 27.75–28.68 Mb) in Angus with evidence of selection. Three QTL databases available online (http://genomes.sapac.edu.au/bovineqtl/index.html, http://www.animalgenome.org/QTLdb/cattle.html, http://www.vetsci.usyd.edu.au/reprogen/QTL_Map/) were used to identify the chromosomal coordinates of published QTL in beef and dairy cattle on BTA19 and 29. Markers within the reported QTL regions were aligned to the third draft of the bovine genome sequence assembly (Btau 3.1) to obtain the approximate position of these QTL in Mb, and these are reported in Table S4. We found agreement between the regions with large allele frequency differences and those that had previously been identified to be harbouring beef or dairy QTL (Figs 2 & 3). Using this approach, we sought regions where Angus has been selected for alleles that have been selected against in Holstein. Such differences in allelic frequencies, however, may arise because of selection, drift or admixture. Although we cannot completely rule out the possibility of allele frequency differences caused by drift or admixture, the finding that there is statistically significant agreement between chromosomal regions having large allele frequency differences with QTL regions provides independent evidence for selection over drift, which is a random process. Our approach does suffer from the fact that when markers are not equally spaced on the chromosome, the five-locus sliding window will not cover the same physical distance, which may affect the correlation between allele frequencies expected within each window and thus the range of breed differences. In addition, permutation tests may disrupt the correlation that is expected to exist between allelic frequencies at neighbouring loci as a result of selection. It is also important to note that the reported QTL peaks are generally quite broad and were reported from different resource populations, which may not have direct relevance to the populations studied here. To address these issues, we utilized an EHH approach, which detects selection by detecting the presence of long-range haplotypes that putatively harbour selected alleles within a population. The chromosome-wide scan detected three regions (44.417–44.514, 61.308–61.355 and 62.017–62.184 Mb) in Holstein and one region (40.444–40.889 Mb) in Angus on BTA19 that showed evidence of selection. On BTA29, we found four regions (11.655–11.739, 29.840–31.096, 31.807–32.078 and 33.693–34.136 Mb) in Holstein and one region (7.767–8.006 Mb) in Angus. In all these regions identified using the EHH approach, we found a core haplotype with the highest frequency and the highest EHH among other core haplotypes at those loci, indicating positive selection (Figs S1–S9). By comparing these regions with the regions identified using the sliding-window approach, we found two regions (11.655–11.739 and 33.693–34.136 Mb) in Holstein and one region (7.767–8.006 Mb) in Angus on BTA29 that were common in both approaches and showed evidence of selection (Table S4).

**Figure 4:**
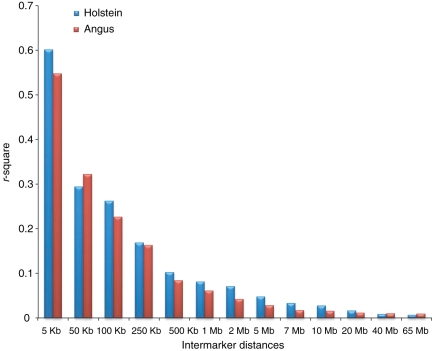
Decay of LD shown by the distribution of *r*^2^ as a function of bins of intermarker distances averaged across both chromosomes.

Graphical representation of the patterns of LD shows regions of high and low LD across the chromosomes in both breeds. A clear difference in the pattern of LD is observed in Angus and Holstein (Figs S10–S13). For instance, on BTA19 from 0 to 2.1 Mb, Holstein shows higher LD than Angus. On BTA29, we see moderate-to-high regions of LD in Holstein at regions 0.54–2.93 and at 37.73–40.82 Mb, which are clearly absent in Angus. However, these regions of higher LD do not align with the regions that possess higher allele frequency differences (results not shown). Although these regions may be expected to show some correlation, the disparity may have arisen because of the use of different sets of SNPs. The LD in these regions could have been generated by complex interactions between biological factors, such as recombination and mutation, and the population’s evolutionary history (Mueller 2004). We observed long-range LD with LD dissipating to background levels at a locus separation of about 20 Mb on both chromosomes (Fig. 4). We cannot make direct comparisons between our study and some of the previous LD studies in cattle (Farnir *et al.* 2000; Tenesa *et al.* 2003; Vallejo *et al.* 2003; Khatkar *et al.* 2006a; Odani *et al.* 2006) that used *D*′ as a measure of LD because we used *r*^2^. We compared our study with that of McKay *et al.* (2007), where LD was also estimated using *r*^2^ and found similar results for Angus and Holstein data. For example, at intermarker distances of 5, 100 and 500 kb in Holstein, the *r*^2^-values in our study were 0.6, 0.26 and 0.1 compared to 0.53, 0.23 and 0.1 in McKay *et al.* (2007). It is important to mention here that the SNPs used in our study and in the McKay *et al.* (2007) study were not the same. McKay *et al.* (2007) used approximately 2670 markers genome-wide, with 54 and 55 markers, respectively for BTA19 and BTA29. However, the animals used by McKay *et al.* (2007) were included in our study. The average *r*^2^-values for BTA19 in McKay *et al.* (2007) were not shown because of the presence of fewer than five informative locus pairs. However, with many more markers on these chromosomes and a larger sample size, our study demonstrates that LD persists over long intermarker distances of up to 20 Mb. It is also important to note that the LD results from our study have come from only two chromosomes, which were not chosen at random and from only two breeds. Therefore, the results from this study may not be representative of the genome as a whole or of all *Bos taurus* breeds. Our study shows that at a physical distance of 100 kb, the average *r*^2^-value is 0.23–0.26. We can assume that any QTL we seek will be at most in the middle of the interval and therefore no more than 50 kb away from any marker. Hence, the average *r*^2^ between these markers and a QTL located at the mid-interval is approximately 0.3. This indicates that there should be an informative marker every 100 kb to achieve a moderate LD (*r*^2^-values ≥ 0.2) for genome-wide association studies. Because the bovine genome is approximately 3 Gb, we would need a minimum of 30 000 evenly spaced and informative markers to perform a whole-genome association study, which agrees with McKay *et al.* (2007) but disagrees with Khatkar *et al.* (2007) and Gautier *et al.* (2007), who have suggested that 75 000–100 000 and 300 000 SNPs, respectively capture most of the LD information within the different cattle breeds based on the identification of haplotype blocks and tag SNPs. Considering the fact that many SNPs may have low MAFs in certain breeds and with the goal of achieving an even spacing across the bovine genome, we concur with McKay *et al.* (2007) who suggested that a 50 000 SNP chip should be sufficient for whole-genome association studies in *Bos taurus* cattle. The information generated from this study has important implications for the design and application of association studies in cattle populations.

## Supporting information

**Table S1** List of the markers used in the LD analysis and their corresponding map positions. The Btau 3.1 positions, RH map positions (Prasad *et al.* 2007) as well as inferred base pair positions (used in the analysis) are provided.

**Table S2** An example of a set of five loci on BTA29 to illustrate how linkage phase of the alleles was inferred using multigenerational pedigree in this study.

**Table S3** List of the frequency of the alleles of 355 and 175 SNP markers on BTA19 and 29 respectively, common in both breeds of cattle, plotted in Figures 2 and 3.

**Table S4** Summary of the comparison of the chromosomal regions showing evidence of selection using the five-locus sliding window and EHH approaches. The chromosomal regions, which showed evidence of selection in both approaches, are highlighted in yellow colour.

**Figure S1** Decay of EHH as a function of distance in Holstein on BTA19. The chromosomal region of 44.417–44.514 Mb was considered as a candidate region, which was defined using markers BTA-45659 and BTA-45683. The sequence and population frequency of each core haplotype are shown after the colons, where A, C, G and T are coded respectively as 1, 2, 3 and 4.

**Figure S2** Decay of EHH as a function of distance in Holstein on BTA19. The chromosomal region of 61.308–61.355 Mb was considered as a candidate region, which was defined using markers BTA-29633 and BTA-29634. The sequence and population frequency of each core haplotype are shown after the colons, where A, C, G and T are coded respectively as 1, 2, 3 and 4.

**Figure S3** Decay of EHH as a function of distance in Holstein on BTA19. The chromosomal region of 62.017–62.184 Mb was considered as a candidate region, which was defined using markers BTA-45659 and BTA-45683. The sequence and population frequency of each core haplotype are shown after the colons, where A, C, G and T are coded respectively as 1, 2, 3 and 4.

**Figure S4** Decay of EHH as a function of distance in Angus on BTA19. The chromosomal region of 40.444–40.889 Mb was considered as a candidate region, which was defined using markers BTA-57053 and BTA-56081. The sequence and population frequency of each core haplotype are shown after the colons, where A, C, G and T are coded respectively as 1, 2, 3 and 4.

**Figure S5** Decay of EHH as a function of distance in Holstein on BTA29. The chromosomal region of 11.655–11.739 Mb was considered as a candidate region, which was defined using markers BTA-112191 and BTA-112193. The sequence and population frequency of each core haplotype are shown after the colons, where A, C, G and T are coded respectively as 1, 2, 3 and 4.

**Figure S6** Decay of EHH as a function of distance in Holstein on BTA29. The chromosomal region of 29.840–31.096 Mb was considered as a candidate region, which was defined using markers BTA-65408 and BTA-04535. The sequence and population frequency of each core haplotype are shown after the colons, where A, C, G and T are coded respectively as 1, 2, 3 and 4.

**Figure S7** Decay of EHH as a function of distance in Holstein on BTA29. The chromosomal region of 31.807–32.078 Mb was considered as a candidate region, which was defined using markers BTA-65574 and BTA-65564. The sequence and population frequency of each core haplotype are shown after the colons, where A, C, G and T are coded respectively as 1, 2, 3 and 4.

**Figure S8** Decay of EHH as a function of distance in Holstein on BTA29. The chromosomal region of 33.693–34.136 Mb was considered as a candidate region, which was defined using markers BTA-65681 and BTA-73109. The sequence and population frequency of each core haplotype are shown after the colons, where A, C, G and T are coded respectively as 1, 2, 3 and 4.

**Figure S9** Decay of EHH as a function of distance in Angus on BTA29. The chromosomal region of 7.767–8.006 Mb was considered as a candidate region, which was defined using markers BTA-06107 and BTA-27538. The sequence and population frequency of each core haplotype are shown after the colons, where A, C, G and T are coded respectively as 1, 2, 3 and 4.

**Figure S10** Pattern of LD estimated using 370 SNP markers on BTA19 in Angus. The horizontal and vertical axes are scaled according to the physical distance between markers. Red represents complete LD and blue represents zero LD for each marker pair.

**Figure S11** Pattern of LD estimated using 367 SNP markers on BTA19 in Holstein. The horizontal and vertical axes are scaled according to the physical distance between markers. Red represents complete LD and blue represents zero LD for each marker pair.

**Figure S12** Pattern of LD estimated using 187 SNP markers on BTA29 in Angus. The horizontal and vertical axes are scaled according to the physical distance between markers. Red represents complete LD and blue represents zero LD for each marker pair.

**Figure S13** Pattern of LD estimated using 179 SNP markers on BTA29 in Holstein. The horizontal and vertical axes are scaled according to the physical distance between markers. Red represents complete LD and blue represents zero LD for each marker pair.
