# Supplementary material for: Linkage disequilibrium and signatures of selection on chromosomes 19 and 29 in beef and dairy cattle
Source: Anim Genet. 2008 Dec;39(6):597–605. doi: 10.1111/j.1365-2052.2008.01772.x (PMC2659388; doi:10.1111/j.1365-2052.2008.01772.x)
Supplement: Supplementary file 1 [file age0039-0597-SD1.pdf]

Supplementary Table 1

BTA	Name	Btau_3_1_Position (bp)	RH_position (cR)	Position used in LD analysis (bp)
19	BTA-25119	No acceptable hits	25.9	357847
19	BTA-46468	594380	50.9	703259
19	BTA-109954	No acceptable hits	77.7	1073541
19	BTA-86613	1673261	124.5	1720153
19	BTA-86615	1673429	124.5	1720321
19	BTA-117829	1815421	134.6	1859700
19	BTA-117833	1815848	139.5	1927400
19	BTA-117835	1816036	143.3	1979903
19	BTA-87957	1880727	143.3	2044594
19	BTA-87958	1880960	145.3	2072227
19	BTA-22161	2148369	167.1	2308735
19	BTA-22160	2159056	172.5	2393913
19	BTA-22155	2159459	174.3	2408214
19	BTA-22153	2388842	190.7	2634805
19	BTA-22149	2446034	190.7	2691997
19	BTA-22150	2446217	190.7	2692180
19	BTA-08011	2558999	196.7	2717704
19	BTA-22143	2560148	198.5	2742573
19	BTA-22140	No acceptable hits	213.6	2951202
19	BTA-22142	No acceptable hits	217.1	2999560
19	BTA-28135	2882099	232.2	3208189
19	BTA-28126	2892590	232.2	3218680
19	BTA-28123	2892860	232.2	3218950
19	BTA-28131	2889022	235.8	3257928
19	BTA-02315	3054038	250.9	3466557
19	BTA-108969	3083810	258.1	3566036
19	BTA-108967	3084132	258.1	3566358
19	BTA-28111	3155340	272.2	3760849
19	BTA-28119	No acceptable hits	283.9	3922502
19	BTA-28112	3155477	287.7	3975004
19	BTA-28106	3157018	287.7	3976545
19	BTA-28107	3157312	287.7	3976839
19	BTA-28108	3157430	287.7	3976957
19	BTA-28104	3159191	287.7	3978718
19	BTA-28153	3161383	287.7	3980910
19	BTA-28152	3173234	287.7	3992761
19	BTA-28120	No acceptable hits	289.4	3998492
19	BTA-28121	3137636	289.4	3999492
19	BTA-28151	3173372	289.4	4035228

19	BTA-46430	3335659	296.6	4097971
19	BTA-46432	3336040	303.6	4194686
19	BTA-46433	3336269	311.9	4309363
19	BTA-13349	3612492	325	4490359
19	BTA-46575	No acceptable hits	329.5	4552533
19	BTA-04223	No acceptable hits	341.1	4712805
19	BTA-44652	4392940	382.6	5286189
19	BTA-44665	4437781	382.6	5331030
19	BTA-44677	4496823	384.5	5357281
19	BTA-44716	4607143	390.5	5395339
19	BTA-44761	4713000	405.7	5605350
19	BTA-06651	4765081	414	5720027
19	BTA-44787	4765466	414	5720412
19	BTA-44793	4791088	420.1	5804307
19	BTA-44815	4836142	423.7	5854047
19	BTA-44817	4840954	434.9	6008792
19	BTA-44888	4950633	437.6	6104108
19	BTA-44889	4950776	440.2	6140031
19	BTA-44893	4955188	440.2	6144443
19	BTA-44928	5047651	447.4	6181498
19	BTA-44927	5048028	447.4	6181875
19	BTA-44930	5048765	447.4	6182612
19	BTA-44965	5162772	449.6	6211894
19	BTA-91865	5270649	456.2	6303083
19	BTA-45143	7271040	467.2	6455064
19	BTA-45487	7227465	479.6	6626389
19	BTA-45490	7227099	487.3	6732776
19	BTA-45492	7223128	489	6756264
19	BTA-45491	7227000	504	6963511
19	BTA-45669	5577645	517.6	7151415
19	BTA-45631	5666813	532.3	7354518
19	BTA-45586	5778246	536.1	7407020
19	BTA-45584	5779444	536.1	7408218
19	BTA-45574	5846561	542.7	7498209
19	BTA-11204	5895385	547.8	7568673
19	BTA-45159	6031524	570.5	7882308
19	BTA-45686	6090492	584.8	8079883
19	BTA-45689	6094309	588.6	8132386
19	BTA-45688	6094357	597.4	8253971
19	BTA-45703	6146724	621.9	8592475
19	BTA-45733	6257000	673.5	9305406

Supplementary Table 1

19	BTA-16709	6791742	715.2	9881554
19	BTA-16718	6881545	724.4	10008665
19	BTA-104142	10956484	747	10320918
19	BTA-45810	10747579	769.1	10626262
19	BTA-46438	10546183	779.3	10767191
19	BTA-46436	10550474	779.3	10771482
19	BTA-46435	10555950	779.3	10776958
19	BTA-46440	10478570	784.8	10843181
19	BTA-45982	10254002	800.7	11062864
19	BTA-13223	10277515	800.7	11086377
19	BTA-24942	7826776	863.3	11927776
19	BTA-24946	7830296	863.3	11931296
19	BTA-46447	8091343	878	12130878
19	BTA-86490	8380176	898.2	12409971
19	BTA-86493	8444361	902.1	12463856
19	BTA-00316	8486477	910	12573006
19	BTA-86498	8486865	913.7	12624127
19	BTA-93463	8663414	924.7	12776108
19	BTA-25637	9463692	979.3	13530489
19	BTA-46509	9757936	1015.9	14036172
19	BTA-97840	11247598	1022.6	14128743
19	BTA-46474	11433573	1043.9	14423034
19	BTA-46456	11853617	1067.6	14750485
19	BTA-46514	12421728	1107.3	15298999
19	BTA-09214	12775626	1131.9	15638885
19	BTA-46564	12838553	1138.1	15724547
19	BTA-46552	12859481	1162.1	16056143
19	BTA-46543	12936368	1181.7	16326946
19	BTA-05909	12963665	1187	16400174
19	BTA-29947	No acceptable hits	1204.5	16641962
19	BTA-46527	13542315	1211.7	16741441
19	BTA-44521	14791021	1222.8	16894804
19	BTA-07806	14692391	1228.1	17101885
19	BTA-44540	14522558	1249.2	17393413
19	BTA-11922	14498062	1263.2	17452990
19	BTA-44552	14453528	1268.9	17531744
19	BTA-44555	14309549	1279.7	17680962
19	BTA-44546	14460882	1279.7	17832295
19	BTA-44561	14180324	1287.6	17941446
19	BTA-44563	14030367	1308	18071969
19	BTA-44565	13927005	1323	18279216

19	BTA-44603	15275858	1368.4	18906485
19	BTA-44594	15359973	1379.5	19059848
19	BTA-44618	15704056	1396	19287820
19	BTA-44616	15732326	1399.3	19333414
19	BTA-13335	15739023	1401.1	19358284
19	BTA-44610	15895042	1422.2	19649812
19	BTA-44495	16561362	1447	20017797
19	BTA-20575	16925471	1460.3	20176220
19	BTA-46586	17123199	1471.4	20329583
19	BTA-46585	17125781	1471.4	20332165
19	BTA-46580	17179402	1471.4	20385786
19	BTA-46576	17183401	1471.4	20389785
19	BTA-46571	17289776	1473.1	20413273
19	BTA-15926	17426048	1480.1	20449787
19	BTA-44631	17544730	1489	20572754
19	BTA-44637	17598825	1494.4	20647363
19	BTA-44638	17602483	1497.9	20695720
19	BTA-44649	17803437	1511.2	20879480
19	BTA-44663	18306459	1530.9	21151665
19	BTA-44669	19992954	1548.3	21392071
19	BTA-07830	19271366	1592.8	22006905
19	BTA-118485	19244408	1594.6	22031775
19	BTA-04414	19361415	1594.6	22148782
19	BTA-44726	19578032	1617.1	22342646
19	BTA-44731	19737675	1624.6	22446270
19	BTA-44751	20261350	1632.1	22549893
19	BTA-44791	21257194	1688.2	23324998
19	BTA-44801	21642551	1703.3	23533627
19	BTA-01578	22221177	1727.9	23873513
19	BTA-44833	22422252	1735.2	23974373
19	BTA-44838	22520003	1747.8	24148461
19	BTA-44845	22530396	1752.7	24216162
19	BTA-115853	22857093	1769.8	24452424
19	BTA-11532	22994444	1779	24579536
19	BTA-44868	23062875	1779	24647967
19	BTA-07396	23642950	1810.1	25009229
19	BTA-108581	24048100	1823.8	25198514
19	BTA-44691	25378411	1855.1	25630971
19	BTA-44690	25378004	1863	25740121
19	BTA-44693	No acceptable hits	1866	25781570
19	BTA-98517	24572906	1924	26582927

19	BTA-20935	24268041	1968.2	27193616
19	BTA-44712	No acceptable hits	1978.5	27335925
19	BTA-14962	27237071	1990.5	27501723
19	BTA-44960	27613513	2017.1	27869242
19	BTA-44964	27762168	2025.2	27981156
19	BTA-44976	27919963	2030.8	28058528
19	BTA-44980	28189564	2045.7	28264394
19	BTA-44981	28207824	2045.7	28282654
19	BTA-44985	28293595	2057.7	28430191
19	BTA-44989	28299387	2057.7	28435983
19	BTA-44990	28303035	2067.6	28566975
19	BTA-01174	28376343	2067.6	28640283
19	BTA-44994	28396324	2075.5	28676125
19	BTA-104726	28456076	2087.8	28846068
19	BTA-67105	29137240	2135.2	29500969
19	BTA-45030	29180085	2158.2	29818749
19	BTA-45023	No acceptable hits	2180.9	30132383
19	BTA-13124	No acceptable hits	2182.6	30155871
19	BTA-45027	No acceptable hits	2182.6	30155871
19	BTA-29349	29493356	2187.7	30226335
19	BTA-106969	29630223	2211.8	30559313
19	BTA-45064	29835781	2217	30631158
19	BTA-45066	29999924	2224.4	30733400
19	BTA-45079	30126442	2231.3	30828734
19	BTA-20635	30064294	2256.1	31171383
19	BTA-45082	30242570	2268.3	31339944
19	BTA-11476	30576168	2282.1	31530612
19	BTA-05960	30794237	2294.6	31703318
19	BTA-17255	31126345	2320.3	32058402
19	BTA-11250	31636221	2354.3	32528162
19	BTA-97038	31641445	2358	32579283
19	BTA-45090	31880392	2378.3	32859758
19	BTA-45036	32554954	2401.7	33183064
19	BTA-45040	32558584	2409.2	33286688
19	BTA-45043	32893554	2423.2	33480119
19	BTA-45047	34013261	2446.4	33800661
19	BTA-45106	34198459	2448.1	33824149
19	BTA-45109	34336058	2455	33919483
19	BTA-45146	35290150	2476.8	34220682
19	BTA-07221	37703198	2488.3	34379572
19	BTA-45369	37840528	2498	34513592

19	BTA-45368	37840572	2498	34513636
19	BTA-45372	37840956	2506	34624124
19	BTA-45375	37841152	2510.4	34684916
19	BTA-45377	37846940	2517.8	34787159
19	BTA-45380	37856356	2525.3	34890782
19	BTA-45379	37856592	2530.7	34965391
19	BTA-45269	37893849	2539	35080068
19	BTA-11992	37891193	2556.4	35320475
19	BTA-45275	37937582	2556.4	35366864
19	BTA-45285	38036458	2576.9	35603713
19	BTA-45288	38061493	2586.1	35755529
19	BTA-45292	38071106	2587.9	35784807
19	BTA-45299	38255512	2597.8	35892478
19	BTA-45304	38305369	2610	36061039
19	BTA-45303	38305433	2612.8	36099725
19	BTA-45302	38305511	2615.9	36142556
19	BTA-45305	No acceptable hits	2619.9	36197822
19	BTA-45314	35539006	2630.1	36338750
19	BTA-45315	35541786	2630.1	36341530
19	BTA-45316	35541603	2633.6	36387108
19	BTA-45318	35649180	2637	36484440
19	BTA-09802	35728534	2644	36530800
19	BTA-45325	35965453	2672	36917661
19	BTA-05437	36271521	2690.7	37176030
19	BTA-45357	36426661	2697.3	37267218
19	BTA-45358	36426545	2699.4	37296233
19	BTA-45356	36426989	2699.4	37296677
19	BTA-45339	36701619	2709.4	37434398
19	BTA-45654	36909022	2715.3	37515915
19	BTA-45350	37204733	2722.6	37616776
19	BTA-45351	37205107	2724.4	37641645
19	BTA-45352	37252173	2729.8	37716254
19	BTA-88705	37321249	2733.3	37767801
19	BTA-45382	38873919	2748.5	37974623
19	BTA-45499	38945217	2755	38064430
19	BTA-45494	39087406	2761.5	38154237
19	BTA-45474	39242304	2772.7	38308982
19	BTA-04699	39335789	2779.7	38405697
19	BTA-45439	40294242	2795.1	38618471
19	BTA-45448	40305196	2803.3	38731766
19	BTA-45457	40473192	2813.5	38872695

19	BTA-45458	40473316	2819.8	38959739
19	BTA-45468	40815820	2836.9	39196000
19	BTA-45470	40884686	2836.9	39264866
19	BTA-45469	40875686	2840.8	39361533
19	BTA-45404	41160181	2870.6	39661616
19	BTA-57050	41395620	2925.4	40418760
19	BTA-57051	41395742	2925.4	40418882
19	BTA-57052	41395973	2925.4	40419113
19	BTA-57053	41396238	2927.2	40443630
19	BTA-55942	41647565	2944.2	40678510
19	BTA-55938	41647926	2950.7	40768317
19	BTA-56081	41842164	2959.4	40888521
19	BTA-45517	No acceptable hits	2974.6	41098531
19	BTA-45521	43831356	2982	41200773
19	BTA-45527	43835302	2987.2	41272619
19	BTA-03390	41925198	3028.6	41844622
19	BTA-45570	41959859	3030.5	41870873
19	BTA-99555	42351680	3086.9	42650123
19	BTA-99554	42351843	3086.9	42650286
19	BTA-45537	43162836	3151.8	43546813
19	BTA-45532	43365613	3160.4	43665635
19	BTA-45661	45198423	3207.6	44317773
19	BTA-45659	45093795	3214.8	44417252
19	BTA-45683	48428056	3221.8	44513967
19	BTA-45684	48423165	3223.3	44534692
19	BTA-45682	48434584	3223.3	44546111
19	BTA-45680	48436977	3226.4	44577523
19	BTA-45676	48528990	3228.7	44609301
19	BTA-02462	45729957	3245.7	44844182
19	BTA-93411	46177067	3258.7	45023796
19	BTA-93414	46180955	3258.7	45027684
19	BTA-45579	46206608	3261.8	45066627
19	BTA-45581	46496129	3275	45249005
19	BTA-45589	46607930	3279.9	45316706
19	BTA-45597	46814933	3284.7	45383025
19	BTA-45615	47303266	3313.6	45782321
19	BTA-45621	47361144	3319.8	45867984
19	BTA-03894	47669239	3338.4	46124970
19	BTA-103899	47734355	3346.3	46234120
19	BTA-45701	48130948	3365.5	46511232
19	BTA-45731	49034896	3372.5	46596113

Supplementary Table 1

19	BTA-45732	49077155	3372.5	46638372
19	BTA-45743	49322386	3379.4	46849705
19	BTA-45737	49442626	3382.8	46896681
19	BTA-45750	49549238	3387.6	46963001
19	BTA-13041	49751808	3432.4	47423720
19	BTA-45906	49754343	3435.8	47470696
19	BTA-45908	49775765	3447.9	47637876
19	BTA-13047	49792874	3449.6	47661364
19	BTA-13045	49793463	3449.6	47661953
19	BTA-45802	50817830	3490.8	48230603
19	BTA-45799	50817928	3493.4	48266526
19	BTA-45795	50821046	3493.4	48269644
19	BTA-45794	50821128	3493.4	48269726
19	BTA-45793	50822025	3496.1	48303831
19	BTA-45770	51407665	3527.6	48739050
19	BTA-45768	51450178	3532.3	48803988
19	BTA-05671	No acceptable hits	3537.3	48873070
19	BTA-91568	55295354	3567	49283420
19	BTA-45875	52236375	3614.4	49938321
19	BTA-45868	52241101	3615.4	49952138
19	BTA-45864	52296802	3619.4	50007404
19	BTA-45860	52533124	3634.2	50211888
19	BTA-45846	52711157	3648.6	50410845
19	BTA-00405	55700010	3655	50499271
19	BTA-04652	52871906	3664.7	50633291
19	BTA-45843	52879881	3671.6	50728625
19	BTA-45829	52921826	3676.1	50790799
19	BTA-45937	53717744	3702.3	51152791
19	BTA-03377	53837212	3710.1	51260560
19	BTA-45954	53958398	3714.4	51319970
19	BTA-45963	54067562	3719.6	51391816
19	BTA-45966	54247921	3724.8	51463662
19	BTA-45979	55147119	3746.7	51766243
19	BTA-07747	54813700	3757.6	51916843
19	BTA-46072	54631221	3771.7	52111655
19	BTA-46037	54290546	3785.8	52306468
19	BTA-46095	56834335	3814.7	52705764
19	BTA-46135	57321380	3837.5	53020780
19	BTA-46121	57514273	3849.1	53181052
19	BTA-46115	57601197	3851.9	53219738
19	BTA-111179	57747983	3871.1	53485014

19	BTA-46256	57887401	3876.4	53558242
19	BTA-46126	No acceptable hits	3886.1	53692262
19	BTA-01709	No acceptable hits	3889.7	53742001
19	BTA-46265	58766556	3964	54768566
19	BTA-46262	58895851	3969.4	54843175
19	BTA-46280	59045077	3977.6	54956471
19	BTA-46281	59052929	3981.7	55013118
19	BTA-46285	59187630	4001	55279776
19	BTA-46292	59377410	4011.5	55424850
19	BTA-46305	59453184	4014.4	55464917
19	BTA-109506	59487290	4017.4	55569770
19	BTA-05874	59610818	4023.6	55592029
19	BTA-77447	59684113	4030	55680455
19	BTA-46306	59453081	4050.1	55958166
19	BTA-46288	59361328	4051.5	55977509
19	BTA-46307	59452716	4051.5	56068897
19	BTA-46313	59462571	4051.5	56078752
19	BTA-46302	59450220	4052.8	56096714
19	BTA-109495	59528681	4057	56154743
19	BTA-109491	59552673	4058.4	56174086
19	BTA-77448	59683956	4061.3	56214154
19	BTA-03306	59922083	4070.1	56234496
19	BTA-46322	59950043	4089.8	56506681
19	BTA-09444	60031335	4104	56702875
19	BTA-84899	60090256	4109	56771957
19	BTA-84891	60159701	4109	56841402
19	BTA-84898	60090311	4112	56882852
19	BTA-84894	60152334	4116.4	56943644
19	BTA-46341	60271950	4132	57089737
19	BTA-46342	60271637	4136	57145003
19	BTA-46348	60310996	4147	57296984
19	BTA-104736	60528281	4164.1	57533246
19	BTA-104738	60528699	4167.1	57574695
19	BTA-104739	60528745	4171.1	57629961
19	BTA-104732	60619700	4224.4	58366380
19	BTA-93880	60795636	4285.3	59207805
19	BTA-46056	60849522	4293.3	59318337
19	BTA-46057	60849890	4293.3	59318705
19	BTA-07437	60862980	4294.8	59339061
19	BTA-46059	60879236	4296.4	59361168
19	BTA-46360	61206306	4312.8	59587758

Supplementary Table 1

19	BTA-46361	61297322	4319.5	59680329
19	BTA-46363	61356465	4326.2	59772899
19	BTA-46364	61366247	4338.5	59942842
19	BTA-05949	61366772	4341.9	59989818
19	BTA-46380	61525711	4367.8	60347665
19	BTA-46381	61526065	4367.8	60348019
19	BTA-05994	61807084	4383.7	60567347
19	BTA-46408	61840399	4388.5	60633666
19	BTA-46409	61840464	4388.5	60633731
19	BTA-46413	61843417	4388.5	60636684
19	BTA-46416	61865210	4391.7	60677879
19	BTA-46407	61840366	4398.2	60767686
19	BTA-46404	61840029	4399.8	60789793
19	BTA-21385	62425783	4407.8	60900325
19	BTA-21380	62416561	4410.1	60932103
19	BTA-07431	62452858	4419.2	61057833
19	BTA-21384	62425919	4424.1	61125533
19	BTA-21181	62359670	4430.7	61216722
19	BTA-29633	62489901	4437.3	61307911
19	BTA-29634	62489797	4440.7	61354887
19	BTA-07433	62452990	4443.9	61399100
19	BTA-07434	62453236	4443.9	61399346
19	BTA-29628	62485848	4443.9	61431958
19	BTA-29635	62489726	4443.9	61435836
19	BTA-12079	62296638	4450.3	61487525
19	BTA-21185	62065513	4467.2	61721024
19	BTA-01614	61960480	4474.4	61820503
19	BTA-105913	No acceptable hits	4482.8	61936561
19	BTA-105515	No acceptable hits	4488.6	62016697
19	BTA-105530	No acceptable hits	4500.7	62183877
19	BTA-105528	No acceptable hits	4508.8	62295790
19	BTA-13718	62877328	4547.4	62829107
19	BTA-46020	63437047	4577.4	63243601
19	BTA-46021	63436861	4579.1	63267089
19	BTA-46024	63432577	4591.4	63437032
29	BTA-65690	6551830	0	0
29	BTA-109603	No acceptable hits	35.3	544086
29	BTA-66450	7239703	62.6	964866
29	BTA-03053	7324685	141.9	2187133
29	BTA-66438	7553282	183.6	2829864
29	BTA-66437	7557431	190.2	2931591

29	BTA-66411	7917898	209	3221359
29	BTA-66407	7967828	213.4	3289177
29	BTA-66158	8298150	237.1	3654470
29	BTA-66134	8358370	269.5	4153857
29	BTA-66472	8403689	291	4485241
29	BTA-66400	8577397	316.4	4876736
29	BTA-66404	8576824	335	5163422
29	BTA-66395	14425067	335	5164422
29	BTA-07370	9587015	346.3	5337591
29	BTA-66525	9589878	348.5	5371500
29	BTA-66550	9725070	357.8	5514843
29	BTA-66565	9878783	364.6	5619653
29	BTA-66570	9880078	366.8	5653562
29	BTA-66587	No acceptable hits	377.8	5823107
29	BTA-66575	10001310	379.8	5853933
29	BTA-66576	10001422	381.9	5886301
29	BTA-66579	10109100	399.8	6162197
29	BTA-66617	8921274	416.8	6424222
29	BTA-117883	9067881	423.2	6522866
29	BTA-105620	9158277	425.7	6561399
29	BTA-105615	9374923	445.7	6869663
29	BTA-105616	9427564	450.3	6940564
29	BTA-105618	9477879	452.2	6969849
29	BTA-24968	No acceptable hits	474.9	7319729
29	BTA-24970	10570960	481.4	7419914
29	BTA-18356	No acceptable hits	492.3	7587918
29	BTA-66634	No acceptable hits	492.3	7588918
29	BTA-06107	No acceptable hits	503.9	7766711
29	BTA-27538	No acceptable hits	519.4	8005616
29	BTA-27534	No acceptable hits	531.6	8193657
29	BTA-120302	No acceptable hits	533.7	8226025
29	BTA-113857	No acceptable hits	590.9	9108660
29	BTA-113862	No acceptable hits	590.9	9109660
29	BTA-113865	No acceptable hits	595.1	9172395
29	BTA-90456	No acceptable hits	606	9340399
29	BTA-70172	11162025	647.1	9973882
29	BTA-105939	11355456	702.5	10827773
29	BTA-105940	11434395	720.4	11103669
29	BTA-105947	11918715	729	11236223
29	BTA-105961	11961509	733.3	11302500
29	BTA-117782	No acceptable hits	749.7	11555276

Supplementary Table 1

29	BTA-112191	11778368	756.2	11655462
29	BTA-112193	11745781	761.6	11738693
29	BTA-16286	No acceptable hits	763.7	11771061
29	BTA-22554	No acceptable hits	771.1	11885119
29	BTA-64906	12267097	797.9	12298192
29	BTA-64902	12331139	797.9	12362234
29	BTA-93929	12432106	810.9	12498564
29	BTA-08572	12491559	823.6	12694312
29	BTA-08585	12494591	834	12854609
29	BTA-08579	12494817	840.1	12948629
29	BTA-64907	13533785	858.7	13235315
29	BTA-12750	13506969	870.5	13417191
29	BTA-08577	12494944	894.2	13782483
29	BTA-08584	12494671	907.5	13987479
29	BTA-64938	13138674	983.1	15152717
29	BTA-64937	13142735	983.1	15156778
29	BTA-64934	13182085	991.6	15283729
29	BTA-64925	13329967	993.8	15317638
29	BTA-64976	14123339	1023.8	15780034
29	BTA-65055	17984263	1158.6	17857734
29	BTA-65056	18042011	1163.1	17927093
29	BTA-16404	18228382	1174.5	18102803
29	BTA-16399	18327908	1178.9	18170621
29	BTA-16409	18380865	1178.9	18223578
29	BTA-16410	18381007	1178.9	18223720
29	BTA-16408	18385172	1178.9	18227885
29	BTA-16406	18421151	1178.9	18263864
29	BTA-106563	18520840	1183.2	18330141
29	BTA-38148	18813901	1205.2	18575989
29	BTA-38149	18814122	1205.2	18576210
29	BTA-38144	18834618	1207.1	18605274
29	BTA-03493	18953172	1213.2	18699294
29	BTA-116569	19101039	1223.7	18861133
29	BTA-65064	22347626	1232	18989062
29	BTA-65068	22326401	1236.2	19053798
29	BTA-09899	No acceptable hits	1244.6	19183269
29	BTA-65072	No acceptable hits	1246.5	19212554
29	BTA-65070	No acceptable hits	1250	19266500
29	BTA-65073	No acceptable hits	1261.3	19440669
29	BTA-26204	19679349	1290.2	19886111
29	BTA-26203	19576768	1307	20145052

29	BTA-26202	19685207	1309.2	20178961
29	BTA-26209	No acceptable hits	1315.8	20280689
29	BTA-61000	No acceptable hits	1332.3	20535006
29	BTA-17015	No acceptable hits	1347.2	20764663
29	BTA-17014	No acceptable hits	1356.7	20911088
29	BTA-65087	19794934	1377.2	21227059
29	BTA-65091	19818653	1404.7	21650922
29	BTA-65104	20192322	1419	21871331
29	BTA-07708	20192592	1421.2	21905240
29	BTA-65111	20337107	1443.5	22248954
29	BTA-65113	20346560	1448.4	22324479
29	BTA-08389	20390911	1452.8	22392297
29	BTA-65147	20706613	1466.6	22604999
29	BTA-65151	20842012	1478.2	22783792
29	BTA-65154	20879747	1491	22981081
29	BTA-65153	20879798	1494	23027321
29	BTA-65157	20889230	1500.7	23130589
29	BTA-65162	20996680	1505.2	23199949
29	BTA-65165	21020932	1505.2	23224201
29	BTA-65224	24083463	1619.5	24961677
29	BTA-12811	24122252	1619.5	25000466
29	BTA-65220	24183498	1630.5	25131223
29	BTA-65388	24397290	1653.9	25491891
29	BTA-65386	24511854	1676.4	25838688
29	BTA-85826	24569830	1680.3	25898800
29	BTA-85843	24603206	1682.3	25929626
29	BTA-85871	24645632	1682.3	25972052
29	BTA-85838	24602780	1693.4	26100713
29	BTA-85869	24640245	1693.4	26138178
29	BTA-65297	24916205	1700.3	26207064
29	BTA-65291	No acceptable hits	1708.8	26338076
29	BTA-65277	No acceptable hits	1714.2	26421307
29	BTA-65293	24919792	1714.2	26422307
29	BTA-65271	25091644	1714.2	26594159
29	BTA-65301	24910888	1716.3	26626527
29	BTA-65296	24916160	1716.3	26631799
29	BTA-65498	24980684	1716.3	26696323
29	BTA-65275	25074152	1716.3	26789791
29	BTA-65272	25087569	1716.3	26803208
29	BTA-65268	25092063	1716.3	26807702
29	BTA-106381	25604485	1739.1	27159123

29	BTA-106382	25604798	1748.4	27302466
29	BTA-106378	25607225	1752.5	27365660
29	BTA-106289	25646652	1752.5	27405087
29	BTA-65467	25684513	1756.6	27468281
29	BTA-90762	25821044	1769.8	27671735
29	BTA-90745	25876637	1774.4	27742636
29	BTA-90754	25926348	1776.6	27776545
29	BTA-90746	25893154	1778.8	27810454
29	BTA-90748	25930615	1778.8	27847915
29	BTA-65531	26183895	1792.7	28062159
29	BTA-65524	26326158	1804.3	28240952
29	BTA-65517	26331428	1808.9	28311852
29	BTA-65515	26338295	1815.7	28416662
29	BTA-65505	26418334	1832.8	28680228
29	BTA-22805	26473417	1835.1	28715678
29	BTA-22801	26478459	1835.1	28720720
29	BTA-10760	26572851	1837.4	28756171
29	BTA-65444	26774637	1844.6	28867146
29	BTA-65427	27175084	1869.9	29257100
29	BTA-65433	27098654	1898.5	29261960
29	BTA-74283	27616714	1911.4	29460790
29	BTA-65408	27845359	1936	29839955
29	BTA-65395	27968227	1956.6	30157467
29	BTA-04535	28872454	2017.5	31096131
29	BTA-66492	32308046	2034.5	31358155
29	BTA-65574	30715403	2063.6	31806680
29	BTA-65570	30866005	2076.3	32002427
29	BTA-65564	30926928	2081.2	32077952
29	BTA-65568	30927204	2086.1	32153477
29	BTA-65555	31077355	2096.5	32313774
29	BTA-65658	31414716	2126.9	32782335
29	BTA-65662	31415195	2128.8	32811620
29	BTA-65717	31467606	2136.5	32930302
29	BTA-65713	31471238	2138.4	32959587
29	BTA-65699	31662505	2148.3	33112178
29	BTA-29794	34310798	2162	33323338
29	BTA-29792	34310594	2166.4	33391156
29	BTA-02252	34063621	2181.4	33622354
29	BTA-65681	No acceptable hits	2186	33693255
29	BTA-73109	29074886	2214.7	34135614
29	BTA-65656	29338358	2242.8	34568725

29	BTA-65646	29623983	2250.8	34692031
29	BTA-65642	29700474	2253	34725940
29	BTA-07368	29830428	2261.6	34858493
29	BTA-99814	29847555	2263.8	34892402
29	BTA-102309	No acceptable hits	2376.9	36635635
29	BTA-65775	33275674	2390.2	36840631
29	BTA-65785	33375735	2395.9	36928486
29	BTA-65879	36022288	2441	37623621
29	BTA-106996	36162872	2446.7	37711476
29	BTA-106994	36182954	2446.7	37731558
29	BTA-65836	36780009	2497.7	38497550
29	BTA-65853	37599641	2539.5	39141821
29	BTA-66030	38343713	2561.7	39483994
29	BTA-65943	39105363	2601.8	40102064
29	BTA-09465	39238587	2610.4	40234617
29	BTA-09466	39238774	2615.2	40308601
29	BTA-65938	39286978	2621.8	40410328
29	BTA-66057	39739339	2635.9	40627654
29	BTA-66045	40105553	2648.6	40823402
29	BTA-66333	41083861	2667.2	41110087
29	BTA-66126	41030087	2669.4	41143996
29	BTA-117001	40841859	2673.5	41207190
29	BTA-116993	40842109	2673.5	41207440
29	BTA-66071	40392915	2692.9	41506206
29	BTA-01521	41312565	2704	41677293
29	BTA-66095	41561582	2715.2	41849921
29	BTA-66106	41637543	2717.1	41879206
29	BTA-66122	41657341	2718.9	41906949
29	BTA-66154	41737998	2722.7	41965520
29	BTA-66215	42372421	2749.1	42372428
