# Supplementary material for: Linkage disequilibrium and signatures of selection on chromosomes 19 and 29 in beef and dairy cattle
Source: Anim Genet. 2008 Dec;39(6):597–605. doi: 10.1111/j.1365-2052.2008.01772.x (PMC2659388; doi:10.1111/j.1365-2052.2008.01772.x)
Supplement: Supplementary file 2 [file age0039-0597-SD2.pdf]

Supplementary Table 2

Generation	Analysis ID	Sex	Animal	Sire	Dam	m	Marker Name	Mat	Pat	pGmx	oGmx	Position_Mb
Great Grandsire	149	M	1775791	1180703	1348259	23	BTA-105620	1	1	0.999800178	0.5	6.561399
						24	BTA-105615	1	1	0.999998976	0.5	6.869663
						25	BTA-105616	3	3	0.999999989	0.5	6.940564
						26	BTA-105618	3	2	1	1	6.969849
						27	BTA-24968	2	1	1	1	7.319729
	Analysis ID	Sex	Animal	Sire	Dam	m	Marker Name	Mat	Pat	pGmx	oGmx	Position_Mb
Grandsire	149	M	2178378	1775791	2094766	23	BTA-105620	1	1	0.999800206	0.5	6.561399
						24	BTA-105615	1	1	0.999998976	0.5	6.869663
						25	BTA-105616	2	3	0.99999999	1	6.940564
						26	BTA-105618	3	3	0.99999964	0.5	6.969849
						27	BTA-24968	2	2	0.999999605	0.5	7.319729
	Analysis ID	Sex	Animal	Sire	Dam	m	Marker Name	Mat	Pat	pGmx	oGmx	Position_Mb
Sire	149	M	2672891	2178378	2672892	23	BTA-105620	3	1	1	1	6.561399
						24	BTA-105615	2	1	1	1	6.869663
						25	BTA-105616	3	2	0.999999146	1	6.940564
						26	BTA-105618	2	3	0.999999206	1	6.969849
						27	BTA-24968	1	2	1	1	7.319729
	Analysis ID	Sex	Animal	Sire	Dam	m	Marker Name	Mat	Pat	pGmx	oGmx	Position_Mb
Progeny	149	M	41276196	2672891	38271010	23	BTA-105620	3	1	0.999999957	1	6.561399
						24	BTA-105615	2	1	0.999999838	1	6.869663
						25	BTA-105616	3	2	0.999999978	1	6.940564
						26	BTA-105618	2	3	0.999999972	1	6.969849
						27	BTA-24968	1	2	0.999999911	1	7.319729

Supplementary Table 2

	Analysis ID	Sex	Animal	Sire	Dam	m	Marker Name	Mat	Pat	pGmx	oGmx	Position_Mb
Progeny	149	M	38580406	2672891	17160148	23	BTA-105620	3	1	0.994974011	1	6.561399
						24	BTA-105615	1	1	0.994776803	0.5	6.869663
						25	BTA-105616	2	2	0.994776812	0.5	6.940564
						26	BTA-105618	3	3	0.99477686	0.5	6.969849
						27	BTA-24968	2	2	0.99477664	0.5	7.319729
	Analysis ID	Sex	Animal	Sire	Dam	m	Marker Name	Mat	Pat	pGmx	oGmx	Position_Mb
Progeny	149	M	38362483	2672891	2413024	23	BTA-105620	1	3	0.994973874	1	6.561399
						24	BTA-105615	2	2	0.994776867	0.5	6.869663
						25	BTA-105616	3	3	0.994776047	0.5	6.940564
						26	BTA-105618	2	2	0.994776099	0.5	6.969849
						27	BTA-24968	1	1	0.994776796	0.5	7.319729
	Analysis ID	Sex	Animal	Sire	Dam	m	Marker Name	Mat	Pat	pGmx	oGmx	Position_Mb
Progeny	149	M	39020869	2672891	39020866	23	BTA-105620	1	3	0.994985821	1	6.561399
						24	BTA-105615	1	2	0.99498172	1	6.869663
						25	BTA-105616	3	3	0.994780538	0.5	6.940564
						26	BTA-105618	2	2	0.994780721	0.5	6.969849
						27	BTA-24968	1	1	0.994665771	0.5	7.319729
