# Supplementary material for: Linkage disequilibrium and signatures of selection on chromosomes 19 and 29 in beef and dairy cattle
Source: Anim Genet. 2008 Dec;39(6):597–605. doi: 10.1111/j.1365-2052.2008.01772.x (PMC2659388; doi:10.1111/j.1365-2052.2008.01772.x)
Supplement: Supplementary file 3 [file age0039-0597-SD3.pdf]

Supplementary Table 3

BTA	SNP_ID	Position (Mb)	Allele	Frequency Holstein	Frequency Angus
19	BTA-25119	0.36	*G*	0.2211838	0.228
19	BTA-46468	0.70	*C*	0.33598726	0.22619048
19	BTA-109954	1.07	*A*	0.45962733	0.50396825
19	BTA-86613	1.72	*G*	0.00310559	0.220
19	BTA-86615	1.72	*G*	0.00155763	0.224
19	BTA-117829	1.86	*A*	0.46283784	0.05833333
19	BTA-117833	1.93	*A*	0.4673913	0.06349206
19	BTA-117835	1.98	*A*	0.4673913	0.06746032
19	BTA-87958	2.07	*G*	0.39440994	0.42063492
19	BTA-22161	2.31	*G*	0.32763975	0.55555556
19	BTA-22160	2.39	*C*	0.15264798	0.3531746
19	BTA-22155	2.41	*G*	0.15372671	0.3531746
19	BTA-22149	2.69	*A*	0.25077882	0.3968254
19	BTA-22150	2.69	*C*	0.25077882	0.3968254
19	BTA-22143	2.74	*C*	0.13043478	0.43253968
19	BTA-22140	2.95	*G*	0.471875	0.43495935
19	BTA-22142	3.00	*A*	0.45652174	0.528
19	BTA-28135	3.21	*G*	0.22857143	0.32051282
19	BTA-28126	3.22	*A*	0.38664596	0.34016393
19	BTA-28131	3.26	*A*	0.38629283	0.44047619
19	BTA-02315	3.47	*A*	0.03738318	0.1125
19	BTA-108969	3.57	*A*	0.64130435	0.272
19	BTA-108967	3.57	*C*	0.64174455	0.28174603
19	BTA-28111	3.76	*G*	0.41352201	0.33057851
19	BTA-28119	3.92	*A*	0.03894081	0.204
19	BTA-28112	3.98	*C*	0.41149068	0.36507937
19	BTA-28106	3.98	*A*	0.40993789	0.36507937
19	BTA-28107	3.98	*A*	0.40993789	0.36507937
19	BTA-28108	3.98	*A*	0.41925466	0.36507937
19	BTA-28104	3.98	*C*	0.03881988	0.20634921
19	BTA-28153	3.98	*A*	0.03881988	0.20634921
19	BTA-28152	3.99	*A*	0.29037267	0.37096774
19	BTA-28121	4.00	*A*	0.03881988	0.19918699
19	BTA-28151	4.04	*C*	0.04037267	0.2
19	BTA-46430	4.10	*A*	0.04037267	0.37301587
19	BTA-46432	4.19	*G*	0.0375	0.31914894
19	BTA-46433	4.31	*C*	0.04037267	0.37301587
19	BTA-46575	4.55	*G*	0.57320872	0.29365079
19	BTA-04223	4.71	*G*	0.25465839	0.59920635

19	BTA-44652	5.29	*C*	0.22897196	0.24603175
19	BTA-44665	5.33	*A*	0.30590062	0.4484127
19	BTA-44677	5.36	*A*	0.38975155	0.32539683
19	BTA-44716	5.40	*A*	0.23364486	0.50793651
19	BTA-44761	5.61	*C*	0.26552795	0.448
19	BTA-06651	5.72	*A*	0.14130435	0.03571429
19	BTA-44787	5.72	*A*	0.14130435	0.03571429
19	BTA-44793	5.80	*G*	0.5390625	0.352
19	BTA-44815	5.85	*A*	0.04347826	0.31746032
19	BTA-44888	6.10	*A*	0.36180124	0.38492063
19	BTA-44889	6.14	*A*	0.50778816	0.40079365
19	BTA-44893	6.14	*A*	0.47975078	0.4
19	BTA-44928	6.18	*G*	0.23136646	0.24596774
19	BTA-44927	6.18	*C*	0.23125	0.26033058
19	BTA-44930	6.18	*G*	0.43375394	0.42460317
19	BTA-44965	6.21	*A*	0.21183801	0.04918033
19	BTA-91865	6.30	*A*	0.08255452	0.12096774
19	BTA-45143	6.46	*C*	0.25931677	0.384
19	BTA-45487	6.63	*A*	0.41025641	0.036
19	BTA-45490	6.73	*A*	0.41352201	0.036
19	BTA-45492	6.76	*G*	0.53416149	0.044
19	BTA-45491	6.96	*A*	0.40566038	0.036
19	BTA-45669	7.15	*A*	0.27329193	0.36290323
19	BTA-45631	7.35	*A*	0.41277259	0.28174603
19	BTA-45586	7.41	*A*	0.20496894	0.27731092
19	BTA-45584	7.41	*A*	0.32763975	0.24206349
19	BTA-45574	7.50	*A*	0.06918239	0.12288136
19	BTA-11204	7.57	*A*	0.18012422	0.692
19	BTA-45686	8.08	*A*	0.53697749	0.37301587
19	BTA-45689	8.13	*A*	0.19937695	0.11507937
19	BTA-45688	8.25	*G*	0.55279503	0.36111111
19	BTA-45703	8.59	*A*	0.32242991	0.1733871
19	BTA-45733	9.31	*C*	0.49378882	0.20247934
19	BTA-16709	9.88	*A*	0.08850932	0.80952381
19	BTA-16718	10.01	*G*	0.07788162	0.084
19	BTA-45810	10.63	*G*	0.15109034	0.6031746
19	BTA-46438	10.77	*A*	0.63707165	0.25403226
19	BTA-46436	10.77	*C*	0.63836478	0.24590164
19	BTA-46435	10.78	*G*	0.68530351	0.25
19	BTA-46440	10.84	*G*	0.11335404	0.07936508
19	BTA-45982	11.06	*C*	0.3757764	0.19444444

19	BTA-13223	11.09	*G*	0.56832298	0.24603175
19	BTA-24942	11.93	*A*	0.21183801	0.08
19	BTA-24946	11.93	*G*	0.209375	0.08
19	BTA-46447	12.13	*C*	0.2734375	0.256
19	BTA-86490	12.41	*A*	0.10248447	0.02380952
19	BTA-86493	12.46	*A*	0.53894081	0.22619048
19	BTA-00316	12.57	*A*	0.10559006	0.11904762
19	BTA-86498	12.62	*G*	0.10559006	0.112
19	BTA-25637	13.53	*A*	0.24451411	0.59166667
19	BTA-46509	14.04	*A*	0.13198758	0.06746032
19	BTA-97840	14.13	*G*	0.35046729	0.31746032
19	BTA-46474	14.42	*A*	0.2305296	0.1468254
19	BTA-46456	14.75	*A*	0.36645963	0.33870968
19	BTA-46514	15.30	*G*	0.43167702	0.26190476
19	BTA-09214	15.64	*A*	0.52492212	0.356
19	BTA-46564	15.72	*A*	0.73125	0.14049587
19	BTA-46552	16.06	*A*	0.73913043	0.19047619
19	BTA-46543	16.33	*G*	0.29326923	0.448
19	BTA-05909	16.40	*G*	0.09968847	0.1468254
19	BTA-29947	16.64	*A*	0.41666667	0.31746032
19	BTA-46527	16.74	*A*	0.08074534	0.2195122
19	BTA-44521	16.89	*C*	0.25931677	0.3902439
19	BTA-07806	17.10	*A*	0.15372671	0.37398374
19	BTA-44540	17.39	*G*	0.07763975	0.108
19	BTA-44552	17.53	*A*	0.21806854	0.24796748
19	BTA-44546	17.83	*C*	0.03115265	0.064
19	BTA-44561	17.94	*A*	0.20560748	0.14634146
19	BTA-44563	18.07	*G*	0.3271028	0.216
19	BTA-44603	18.91	*A*	0.39150943	0.0515873
19	BTA-44594	19.06	*A*	0.31289308	0.11111111
19	BTA-44616	19.33	*C*	0.29439252	0.16532258
19	BTA-13335	19.36	*A*	0.31987578	0.40909091
19	BTA-44610	19.65	*A*	0.0576324	0.15447154
19	BTA-20575	20.18	*C*	0.2826087	0.59126984
19	BTA-46586	20.33	*A*	0.14797508	0.228
19	BTA-46580	20.39	*C*	0.52950311	0.36904762
19	BTA-46576	20.39	*G*	0.55362776	0.36440678
19	BTA-46571	20.41	*C*	0.121875	0.09677419
19	BTA-15926	20.45	*C*	0.47663551	0.168
19	BTA-44631	20.57	*C*	0.30279503	0.596
19	BTA-44637	20.65	*C*	0.6242236	0.21825397

19	BTA-44638	20.70	*A*	0.23447205	0.692
19	BTA-44649	20.88	*A*	0.4578125	0.14516129
19	BTA-44669	21.39	*A*	0.1728972	0.2
19	BTA-07830	22.01	*A*	0.40838509	0.572
19	BTA-118485	22.03	*G*	0.5576324	0.404
19	BTA-04414	22.15	*G*	0.23208723	0.44583333
19	BTA-44726	22.34	*A*	0.46118012	0.34126984
19	BTA-44731	22.45	*G*	0.11645963	0.37704918
19	BTA-44751	22.55	*G*	0.1163522	0.17355372
19	BTA-44791	23.32	*A*	0.70962733	0.07936508
19	BTA-44801	23.53	*A*	0.2046875	0.204
19	BTA-01578	23.87	*A*	0.41304348	0.224
19	BTA-44833	23.97	*G*	0.24841772	0.15079365
19	BTA-44838	24.15	*A*	0.36137072	0.49193548
19	BTA-44845	24.22	*A*	0.36645963	0.504
19	BTA-115853	24.45	*G*	0.11801242	0.11111111
19	BTA-44868	24.65	*G*	0.17601246	0.208
19	BTA-07396	25.01	*G*	0.79595016	0.07142857
19	BTA-108581	25.20	*G*	0.2189441	0.7804878
19	BTA-44691	25.63	*G*	0.0310559	0.44444444
19	BTA-44690	25.74	*G*	0.0326087	0.452
19	BTA-44693	25.78	*A*	0.32453416	0.39285714
19	BTA-98517	26.58	*A*	0.234375	0.328
19	BTA-44712	27.34	*A*	0.28504673	0.02380952
19	BTA-14962	27.50	*A*	0.28881988	0.60714286
19	BTA-44960	27.87	*G*	0.09161491	0.11904762
19	BTA-44964	27.98	*A*	0.30685358	0.10714286
19	BTA-44976	28.06	*A*	0.24299065	0.29435484
19	BTA-44985	28.43	*G*	0.36760125	0.112
19	BTA-44989	28.44	*A*	0.06386293	0.12301587
19	BTA-44990	28.57	*T*	0.26168224	0.492
19	BTA-01174	28.64	*A*	0.12772586	0.0515873
19	BTA-104726	28.85	*A*	0.40372671	0.16129032
19	BTA-67105	29.50	*T*	0.77484472	0.14285714
19	BTA-45030	29.82	*A*	0.53571429	0.38095238
19	BTA-45023	30.13	*A*	0.1588785	0.46825397
19	BTA-13124	30.16	*A*	0.55625	0.03174603
19	BTA-45027	30.16	*G*	0.4068323	0.02777778
19	BTA-29349	30.23	*A*	0.44099379	0.37398374
19	BTA-106969	30.56	*A*	0.22413793	0.052
19	BTA-45064	30.63	*A*	0.23417722	0.06147541

19	BTA-45079	30.83	*A*	0.09190031	0.04365079
19	BTA-20635	31.17	*G*	0.0931677	0.04365079
19	BTA-45082	31.34	*C*	0.10403727	0.068
19	BTA-05960	31.70	*C*	0.0015528	0.23809524
19	BTA-11250	32.53	*A*	0.52795031	0.25396825
19	BTA-97038	32.58	*G*	0.52795031	0.24796748
19	BTA-45090	32.86	*G*	0.47943038	0.32142857
19	BTA-45036	33.18	*G*	0.45440252	0.52777778
19	BTA-45040	33.29	*A*	0.54361371	0.43253968
19	BTA-45047	33.80	*G*	0.40031153	0.452
19	BTA-45106	33.82	*G*	0.38244514	0.368
19	BTA-45109	33.92	*A*	0.0326087	0.148
19	BTA-45146	34.22	*A*	0.19409938	0.424
19	BTA-07221	34.38	*T*	0.27018634	0.624
19	BTA-45368	34.51	*A*	0.05745342	0.08730159
19	BTA-45372	34.62	*G*	0.06170886	0.14344262
19	BTA-45375	34.68	*C*	0.15238095	0.408
19	BTA-45377	34.79	*G*	0.16037736	0.42460317
19	BTA-45380	34.89	*A*	0.1890625	0.54365079
19	BTA-45379	34.97	*G*	0.05607477	0.08333333
19	BTA-45269	35.08	*C*	0.21226415	0.22177419
19	BTA-11992	35.32	*A*	0.2211838	0.25
19	BTA-45275	35.37	*G*	0.33800623	0.45634921
19	BTA-45285	35.60	*C*	0.1890625	0.21428571
19	BTA-45288	35.76	*A*	0.16199377	0.46031746
19	BTA-45292	35.78	*A*	0.48913043	0.3015873
19	BTA-45299	35.89	*G*	0.4052795	0.32539683
19	BTA-45304	36.06	*G*	0.22981366	0.10483871
19	BTA-45303	36.10	*A*	0.22981366	0.10714286
19	BTA-45302	36.14	*A*	0.22897196	0.11507937
19	BTA-45305	36.20	*A*	0.19875776	0.09920635
19	BTA-45314	36.34	*G*	0.37732919	0.46
19	BTA-45315	36.34	*T*	0.32608696	0.46370968
19	BTA-45316	36.39	*C*	0.32608696	0.468
19	BTA-09802	36.53	*G*	0.36645963	0.6097561
19	BTA-45325	36.92	*A*	0.10714286	0.40322581
19	BTA-45358	37.30	*A*	0.24299065	0.26209677
19	BTA-45339	37.43	*T*	0.30288462	0.03719008
19	BTA-45654	37.52	*G*	0.2859375	0.16666667
19	BTA-45350	37.62	*A*	0.0578125	0.20661157
19	BTA-45351	37.64	*A*	0.45015576	0.23412698

19	BTA-45352	37.72	*A*	0.40967742	0.21370968
19	BTA-88705	37.77	*G*	0.54037267	0.26587302
19	BTA-45382	37.97	*T*	0.540625	0.43650794
19	BTA-45494	38.15	*G*	0.03881988	0.144
19	BTA-45474	38.31	*A*	0.4109375	0.40873016
19	BTA-04699	38.41	*A*	0.29127726	0.6097561
19	BTA-45439	38.62	*C*	0.12461059	0.29761905
19	BTA-45448	38.73	*C*	0.59190031	0.1031746
19	BTA-45457	38.87	*C*	0.27760252	0.09756098
19	BTA-45458	38.96	*G*	0.44254658	0.38492063
19	BTA-45470	39.26	*G*	0.09937888	0.11111111
19	BTA-45469	39.36	*A*	0.2476489	0.57438017
19	BTA-45404	39.66	*C*	0.03738318	0.156
19	BTA-57050	40.42	*A*	0.52044025	0.47619048
19	BTA-57051	40.42	*A*	0.52484472	0.46825397
19	BTA-57052	40.42	*A*	0.52492212	0.39754098
19	BTA-57053	40.44	*G*	0.52484472	0.46825397
19	BTA-56081	40.89	*G*	0.49688474	0.36904762
19	BTA-45517	41.10	*A*	0.61956522	0.30487805
19	BTA-45521	41.20	*A*	0.31987578	0.44214876
19	BTA-45527	41.27	*C*	0.31931464	0.4233871
19	BTA-03390	41.84	*G*	0.61320755	0.34920635
19	BTA-45570	41.87	*G*	0.4470405	0.504
19	BTA-99555	42.65	*G*	0.4968652	0.42741935
19	BTA-99554	42.65	*G*	0.50626959	0.488
19	BTA-45537	43.55	*G*	0.41459627	0.3015873
19	BTA-45532	43.67	*A*	0.33489097	0.4484127
19	BTA-45661	44.32	*A*	0.25700935	0.012
19	BTA-45659	44.42	*A*	0.6728972	0.30081301
19	BTA-45683	44.51	*A*	0.41588785	0.1733871
19	BTA-45684	44.53	*A*	0.11838006	0.4375
19	BTA-45682	44.55	*A*	0.03571429	0.40322581
19	BTA-45680	44.58	*A*	0.41744548	0.16666667
19	BTA-45676	44.61	*G*	0.51552795	0.38617886
19	BTA-45675	44.64	*A*	0.00931677	0.20634921
19	BTA-02462	44.84	*A*	0.41277259	0.42857143
19	BTA-93411	45.02	*C*	0.4068323	0.488
19	BTA-93414	45.03	*C*	0.4068323	0.484
19	BTA-45579	45.07	*A*	0.0947205	0.07142857
19	BTA-45581	45.25	*G*	0.6109375	0.34920635
19	BTA-45589	45.32	*G*	0.37111801	0.375

19	BTA-45597	45.38	*G*	0.42138365	0.348
19	BTA-45615	45.78	*A*	0.1242236	0.24206349
19	BTA-45621	45.87	*G*	0.49688474	0.5
19	BTA-03894	46.12	*A*	0.38198758	0.09126984
19	BTA-103899	46.23	*G*	0.19626168	0.68650794
19	BTA-45701	46.51	*A*	0.37147335	0.28174603
19	BTA-45731	46.60	*A*	0.3203125	0.23015873
19	BTA-45743	46.85	*A*	0.23987539	0.01190476
19	BTA-45737	46.90	*A*	0.31055901	0.14285714
19	BTA-45750	46.96	*G*	0.215625	0.0952381
19	BTA-13041	47.42	*A*	0.13819876	0.3699187
19	BTA-45908	47.64	*A*	0.13819876	0.38888889
19	BTA-13047	47.66	*A*	0.16925466	0.39516129
19	BTA-13045	47.66	*A*	0.16925466	0.3968254
19	BTA-45802	48.23	*A*	0.27639752	0.488
19	BTA-45799	48.27	*G*	0.49534161	0.31746032
19	BTA-45795	48.27	*A*	0.27570093	0.488
19	BTA-45794	48.27	*G*	0.49534161	0.31746032
19	BTA-45793	48.30	*G*	0.49688474	0.31746032
19	BTA-45770	48.74	*G*	0.4625	0.21825397
19	BTA-45768	48.80	*A*	0.21428571	0.15853659
19	BTA-05671	48.87	*A*	0.16149068	0.05416667
19	BTA-91568	49.28	*A*	0.27018634	0.16
19	BTA-45875	49.94	*A*	0.18322981	0.16269841
19	BTA-45868	49.95	*G*	0.2890625	0.18292683
19	BTA-45864	50.01	*G*	0.04192547	0.05952381
19	BTA-04652	50.63	*G*	0.36956522	0.152
19	BTA-45843	50.73	*A*	0.05279503	0.15079365
19	BTA-45937	51.15	*A*	0.10747664	0.432
19	BTA-03377	51.26	*G*	0.39184953	0.2
19	BTA-45954	51.32	*A*	0.084375	0.57142857
19	BTA-45963	51.39	*C*	0.21118012	0.4484127
19	BTA-45966	51.46	*C*	0.18495298	0.076
19	BTA-45979	51.77	*A*	0.23089172	0.108
19	BTA-07747	51.92	*A*	0.053125	0.33467742
19	BTA-46072	52.11	*G*	0.4328125	0.32142857
19	BTA-46037	52.31	*G*	0.20807453	0.05555556
19	BTA-46095	52.71	*A*	0.51708075	0.13095238
19	BTA-46135	53.02	*G*	0.44080997	0.46747967
19	BTA-46121	53.18	*A*	0.44670846	0.47177419
19	BTA-46115	53.22	*A*	0.20031056	0.39285714

19	BTA-46256	53.56	*T*	0.14285714	0.11904762
19	BTA-46126	53.69	*C*	0.05607477	0.40650407
19	BTA-01709	53.74	*A*	0.05329154	0.41803279
19	BTA-46262	54.84	*A*	0.27329193	0.45121951
19	BTA-46280	54.96	*A*	0.52180685	0.27272727
19	BTA-46281	55.01	*G*	0.15109034	0.37301587
19	BTA-46285	55.28	*A*	0.3140625	0.03174603
19	BTA-46292	55.42	*C*	0.50319489	0.112
19	BTA-46305	55.46	*A*	0.1863354	0.42857143
19	BTA-109506	55.57	*A*	0.23291925	0.29098361
19	BTA-05874	55.59	*C*	0.16614907	0.3452381
19	BTA-77447	55.68	*A*	0.31152648	0.36666667
19	BTA-46306	55.96	*G*	0.1863354	0.428
19	BTA-46307	56.07	*A*	0.21118012	0.22357724
19	BTA-46313	56.08	*G*	0.67445483	0.23412698
19	BTA-46302	56.10	*A*	0.23757764	0.6468254
19	BTA-109495	56.15	*A*	0.22274143	0.308
19	BTA-109491	56.17	*A*	0.23447205	0
19	BTA-77448	56.21	*C*	0.6828125	0.31147541
19	BTA-03306	56.23	*A*	0.3265625	0.23333333
19	BTA-46322	56.51	*G*	0.3734375	0.568
19	BTA-09444	56.70	*A*	0.159375	0.44308943
19	BTA-84899	56.77	*G*	0.13043478	0.264
19	BTA-84891	56.84	*C*	0.2609375	0.4484127
19	BTA-84898	56.88	*A*	0.13043478	0.264
19	BTA-84894	56.94	*A*	0.31987578	0.38
19	BTA-46341	57.09	*A*	0.23676012	0.064
19	BTA-46342	57.15	*A*	0.23913043	0.06349206
19	BTA-46348	57.30	*C*	0.35093168	0.33730159
19	BTA-104738	57.57	*G*	0.10869565	0.144
19	BTA-104739	57.63	*G*	0.10869565	0.144
19	BTA-104732	58.37	*A*	0.22049689	0.004
19	BTA-93880	59.21	*C*	0.4378882	0.292
19	BTA-46056	59.32	*G*	0.71583851	0.136
19	BTA-07437	59.34	*G*	0.57763975	0.11904762
19	BTA-46059	59.36	*A*	0.25310559	0
19	BTA-46360	59.59	*G*	0.6257764	0.05952381
19	BTA-46361	59.68	*G*	0.00621118	0.36904762
19	BTA-46363	59.77	*A*	0.10403727	0.20634921
19	BTA-46364	59.94	*C*	0.14440994	0.28174603
19	BTA-05949	59.99	*G*	0.45807453	0.5

19	BTA-46380	60.35	*G*	0.22826087	0.028
19	BTA-46381	60.35	*T*	0.23557692	0.04435484
19	BTA-05994	60.57	*G*	0.4052795	0.48387097
19	BTA-46408	60.63	*A*	0.01397516	0.46428571
19	BTA-46409	60.63	*A*	0.02795031	0.476
19	BTA-46413	60.64	*G*	0.01242236	0.19047619
19	BTA-46416	60.68	*G*	0.02795031	0.43548387
19	BTA-46407	60.77	*A*	0.01397516	0.46341463
19	BTA-46404	60.79	*A*	0.02795031	0.47619048
19	BTA-21385	60.90	*G*	0.14330218	0.392
19	BTA-21380	60.93	*G*	0.14641745	0.20634921
19	BTA-07431	61.06	*G*	0.42236025	0.332
19	BTA-21181	61.22	*A*	0.24378882	0.30952381
19	BTA-29633	61.31	*C*	0.35109718	0.35714286
19	BTA-29634	61.35	*C*	0.35248447	0.36178862
19	BTA-07433	61.40	*C*	0.42236025	0.32539683
19	BTA-07434	61.40	*A*	0.38785047	0.24206349
19	BTA-29628	61.43	*G*	0.42080745	0.37698413
19	BTA-29635	61.44	*A*	0.42056075	0.37301587
19	BTA-21185	61.72	*G*	0.15062112	0.03571429
19	BTA-01614	61.82	*A*	0.03726708	0.228
19	BTA-105913	61.94	*A*	0.21316614	0.03571429
19	BTA-105515	62.02	*G*	0.46118012	0.3266129
19	BTA-105530	62.18	*G*	0.68167702	0.16666667
19	BTA-105528	62.30	*C*	0.28416149	0.49206349
19	BTA-13718	62.83	*A*	0.14596273	0.5
19	BTA-46020	63.24	*A*	0.00940439	0.376
19	BTA-46021	63.27	*G*	0.14018692	0.8
19	BTA-46024	63.44	*G*	0.23602484	0.51219512
29	BTA-65690	0	*G*	0.22981366	0.3015873
29	BTA-109603	0.54	*A*	0.50934579	0.25
29	BTA-66450	0.96	*G*	0.26242236	0.17063492
29	BTA-03053	2.19	*A*	0.18478261	0.104
29	BTA-66438	2.83	*A*	0.0576324	0.32142857
29	BTA-66437	2.93	*G*	0.64797508	0.208
29	BTA-66411	3.22	*G*	0.52018634	0.35772358
29	BTA-66407	3.29	*A*	0.05590062	0.28
29	BTA-66134	4.15	*A*	0.23831776	0.24206349
29	BTA-66472	4.49	*C*	0.37850467	0.58264463
29	BTA-66400	4.88	*A*	0.27484472	0.08730159
29	BTA-66404	5.16	*C*	0.2734375	0.084

29	BTA-66395	5.16	*G*	0.45031056	0.2
29	BTA-07370	5.34	*G*	0.44875776	0.404
29	BTA-66525	5.37	*A*	0.26863354	0.336
29	BTA-66550	5.51	*A*	0.45768025	0.47131148
29	BTA-66587	5.82	*A*	0.1863354	0.676
29	BTA-66575	5.85	*G*	0.44099379	0.02380952
29	BTA-66576	5.89	*G*	0.4423676	0.028
29	BTA-66579	6.16	*C*	0.12267081	0.62601626
29	BTA-66617	6.42	*A*	0.14330218	0.2375
29	BTA-117883	6.52	*G*	0.43925234	0.3902439
29	BTA-105620	6.56	*G*	0.23913043	0.204
29	BTA-105615	6.87	*C*	0.2921875	0.11507937
29	BTA-105616	6.94	*C*	0.17701863	0.52
29	BTA-105618	6.97	*G*	0.18095238	0.28629032
29	BTA-24968	7.32	*C*	0.24534161	0.3531746
29	BTA-18356	7.59	*G*	0.29192547	0.388
29	BTA-66634	7.59	*A*	0.48757764	0.15983607
29	BTA-06107	7.77	*G*	0.33074534	0.52439024
29	BTA-27538	8.01	*G*	0.196875	0.552
29	BTA-27534	8.19	*G*	0.17857143	0.46428571
29	BTA-120302	8.23	*A*	0.2171875	0.46311475
29	BTA-113862	9.11	*T*	0.25236593	0.29674797
29	BTA-113865	9.17	*A*	0.2492163	0.2875
29	BTA-70172	9.97	*C*	0.46573209	0.53174603
29	BTA-105939	10.83	*A*	0.54037267	0.41269841
29	BTA-105940	11.10	*T*	0.47819315	0.444
29	BTA-105947	11.24	*G*	0.1609375	0.08730159
29	BTA-117782	11.56	*G*	0.3121118	0.13492063
29	BTA-112191	11.66	*G*	0.53144654	0.36904762
29	BTA-112193	11.74	*G*	0.3431677	0.49593496
29	BTA-16286	11.77	*G*	0.31308411	0.26666667
29	BTA-22554	11.89	*A*	0.14485981	0.1547619
29	BTA-64906	12.30	*C*	0.42367601	0.20731707
29	BTA-64902	12.36	*A*	0.15062112	0.14285714
29	BTA-93929	12.50	*C*	0.29503106	0.35714286
29	BTA-08572	12.69	*G*	0.47507788	0.23809524
29	BTA-08585	12.85	*G*	0.475	0.236
29	BTA-08579	12.95	*A*	0.46865204	0.236
29	BTA-08577	13.78	*G*	0.46964856	0.23170732
29	BTA-08584	13.99	*G*	0.47663551	0.236
29	BTA-64938	15.15	*T*	0.0576324	0.05952381

29	BTA-64937	15.16	*C*	0.05900621	0.05952381
29	BTA-64934	15.28	*G*	0.29439252	0.23015873
29	BTA-64925	15.32	*G*	0.07320872	0.044
29	BTA-65056	17.93	*A*	0.11024845	0.38888889
29	BTA-16404	18.10	*A*	0.190625	0.29761905
29	BTA-16399	18.17	*A*	0.51708075	0.09920635
29	BTA-16409	18.22	*G*	0.27570093	0.48770492
29	BTA-16410	18.22	*T*	0.2765625	0.48790323
29	BTA-16408	18.23	*C*	0.5734375	0.30081301
29	BTA-16406	18.26	*A*	0.0947205	0.10714286
29	BTA-38148	18.58	*A*	0.08540373	0.10483871
29	BTA-38149	18.58	*A*	0.08385093	0.11111111
29	BTA-38144	18.61	*G*	0.02018634	0.34920635
29	BTA-03493	18.70	*G*	0.24689441	0.03658537
29	BTA-116569	18.86	*A*	0.44565217	0.44444444
29	BTA-65064	18.99	*G*	0.13239875	0.50396825
29	BTA-65068	19.05	*A*	0.0703125	0.25
29	BTA-09899	19.18	*A*	0.06583072	0.36111111
29	BTA-65070	19.27	*C*	0.23447205	0.376
29	BTA-65073	19.44	*G*	0.23291925	0.375
29	BTA-26204	19.89	*A*	0.22981366	0.0952381
29	BTA-26203	20.15	*A*	0.03571429	0.31818182
29	BTA-26202	20.18	*C*	0.0621118	0.10080645
29	BTA-26209	20.28	*G*	0.36335404	0.1031746
29	BTA-61000	20.54	*A*	0.29127726	0.45238095
29	BTA-17015	20.76	*C*	0.2788162	0.05555556
29	BTA-17014	20.91	*A*	0.28056426	0.05462185
29	BTA-65087	21.23	*A*	0.24378882	0.17460317
29	BTA-65091	21.65	*A*	0.23447205	0.20491803
29	BTA-07708	21.91	*G*	0.32608696	0.284
29	BTA-65111	22.25	*A*	0.14596273	0.12301587
29	BTA-65113	22.32	*A*	0.0843949	0.1468254
29	BTA-08389	22.39	*G*	0.3109375	0.1031746
29	BTA-65147	22.60	*G*	0.19099379	0.26190476
29	BTA-65151	22.78	*G*	0.23824451	0.2804878
29	BTA-65153	23.03	*A*	0.2165109	0.28
29	BTA-65157	23.13	*G*	0.21495327	0.28571429
29	BTA-65162	23.20	*G*	0.35869565	0.01587302
29	BTA-65165	23.22	*A*	0.0984375	0.65725806
29	BTA-65224	24.96	*A*	0.27570093	0.09920635
29	BTA-12811	25.00	*A*	0.17236025	0.056

29	BTA-65220	25.13	*G*	0.55279503	0.10245902
29	BTA-65388	25.49	*T*	0.35046729	0.33333333
29	BTA-85843	25.93	*A*	0.22327044	0.17886179
29	BTA-85871	25.97	*C*	0.35093168	0.31048387
29	BTA-85838	26.10	*A*	0.21962617	0.19512195
29	BTA-65297	26.21	*G*	0.60714286	0.35483871
29	BTA-65291	26.34	*A*	0.07142857	0.32539683
29	BTA-65277	26.42	*G*	0.39252336	0.16532258
29	BTA-65293	26.42	*G*	0.59621451	0.3531746
29	BTA-65301	26.63	*A*	0.39906832	0.2804878
29	BTA-65296	26.63	*A*	0.40498442	0.28688525
29	BTA-65498	26.70	*A*	0.2515528	0.1984127
29	BTA-65275	26.79	*G*	0.38975155	0.10887097
29	BTA-65272	26.80	*A*	0.38975155	0.11290323
29	BTA-65268	26.81	*A*	0.3894081	0.11904762
29	BTA-106381	27.16	*A*	0.12267081	0.136
29	BTA-106382	27.30	*G*	0.31677019	0.13306452
29	BTA-106378	27.37	*G*	0.31832298	0.1300813
29	BTA-65467	27.47	*A*	0.1466877	0.28629032
29	BTA-90762	27.67	*A*	0.0609375	0.05555556
29	BTA-90745	27.74	*T*	0.12111801	0.19047619
29	BTA-90754	27.78	*A*	0.51397516	0.42063492
29	BTA-90746	27.81	*A*	0.14751553	0.37301587
29	BTA-90748	27.85	*C*	0.2515528	0.19444444
29	BTA-65531	28.06	*C*	0.17236025	0.304
29	BTA-65524	28.24	*G*	0.27018634	0.63492063
29	BTA-65517	28.31	*A*	0.04503106	0.436
29	BTA-65515	28.42	*G*	0.48757764	0.348
29	BTA-65505	28.68	*A*	0.41459627	0.19354839
29	BTA-22805	28.72	*G*	0.605919	0.25
29	BTA-22801	28.72	*A*	0.44409938	0.23015873
29	BTA-10760	28.76	*G*	0.41588785	0.424
29	BTA-65444	28.87	*A*	0.41455696	0.38306452
29	BTA-65427	29.26	*T*	0.03416149	0.19354839
29	BTA-74283	29.46	*A*	0.42080745	0.41666667
29	BTA-65408	29.84	*A*	0.45341615	0.53174603
29	BTA-04535	31.10	*A*	0.41925466	0.424
29	BTA-66492	31.36	*A*	0.228125	0.17063492
29	BTA-65574	31.81	*G*	0.53582555	0.375
29	BTA-65564	32.08	*A*	0.42056075	0.48
29	BTA-65568	32.15	*A*	0.4184953	0.47560976

29	BTA-65555	32.31	*G*	0.38785047	0.52
29	BTA-65658	32.78	*G*	0.75776398	0.07142857
29	BTA-65662	32.81	*C*	0.25310559	0.03174603
29	BTA-65717	32.93	*G*	0.18322981	0.22222222
29	BTA-65713	32.96	*A*	0.45638629	0.26190476
29	BTA-65699	33.11	*G*	0.36956522	0.484
29	BTA-29794	33.32	*G*	0.29813665	0.00396825
29	BTA-02252	33.62	*A*	0.32398754	0.336
29	BTA-65681	33.69	*A*	0.33163265	0.00813008
29	BTA-73109	34.14	*T*	0.44654088	0.23966942
29	BTA-65656	34.57	*G*	0.54984424	0.28968254
29	BTA-65646	34.69	*C*	0	0.388
29	BTA-65642	34.73	*A*	0.19565217	0.76984127
29	BTA-07368	34.86	*A*	0.22741433	0.15079365
29	BTA-99814	34.89	*A*	0.24610592	0.48015873
29	BTA-102309	36.64	*A*	0.46261682	0.37698413
29	BTA-65775	36.84	*C*	0.20031056	0.42741935
29	BTA-65785	36.93	*A*	0.24068323	0.404
29	BTA-65879	37.62	*A*	0.36490683	0.23809524
29	BTA-106996	37.71	*A*	0.1568323	0.34016393
29	BTA-106994	37.73	*G*	0.17236025	0.58730159
29	BTA-65836	38.50	*G*	0.04503106	0.056
29	BTA-66030	39.48	*A*	0.19875776	0.216
29	BTA-65943	40.10	*G*	0.15217391	0.092
29	BTA-09465	40.23	*G*	0.16304348	0.11904762
29	BTA-09466	40.31	*A*	0.16304348	0.09126984
29	BTA-65938	40.41	*A*	0.16349206	0.08333333
29	BTA-66057	40.63	*A*	0.27258567	0.10714286
29	BTA-66045	40.82	*G*	0.42946708	0.43852459
29	BTA-66333	41.11	*A*	0.31464174	0.364
29	BTA-66126	41.14	*A*	0.25700935	0.348
29	BTA-117001	41.21	*G*	0.06896552	0.47580645
29	BTA-116993	41.21	*C*	0.0015528	0.29761905
29	BTA-66071	41.51	*G*	0.63551402	0.22619048
29	BTA-01521	41.68	*A*	0.14873418	0.16269841
29	BTA-66095	41.85	*A*	0.14485981	0.08
29	BTA-66106	41.88	*A*	0.18429487	0.14224138
29	BTA-66122	41.91	*G*	0.4390625	0.27777778
29	BTA-66154	41.97	*A*	0.42628205	0.132
29	BTA-66215	42.37	*G*	0.14906832	0.09126984
