# Supplementary material for: Linkage disequilibrium and signatures of selection on chromosomes 19 and 29 in beef and dairy cattle
Source: Anim Genet. 2008 Dec;39(6):597–605. doi: 10.1111/j.1365-2052.2008.01772.x (PMC2659388; doi:10.1111/j.1365-2052.2008.01772.x)
Supplement: Supplementary file 4 [file age0039-0597-SD4.pdf]

Supplementary Table 4

Five locus sliding window results

BTA	Dairy/Beef	Regions showing evidence of selection (Mb)	Trait	QTL peak (cM)	Markers	Position (cM)	Position(Mbp,Btau_3.1)	Database	Reference
19	Dairy	6.18-7.35	Stature	2.7	Centromere-*BM6000*	0-5.35	0-4.45	Iowa State University	Ashwell et al. 2005
		9.88-11.93	-	-	-	-	-	-	-
		14.75-17.10	SCS	50	*DIK2657*	43.6	22.29	Australian database	Bennewitz et al. 2003
19	Dairy	28.64-30.83	Milk fat	61	*BMS2389*	61.6	31.80	Australian database	Bennewitz et al. 2003
19	Dairy	57.15-59.68	Milk fat	77.68-86.01	*DIK5208-IDVGA-44*	77.68-86.01	52.26-55.88	Bovine QTL viewer	Bennewitz et al. 2003
			Fat%	93	*ETH3*	92	54.52	Australian database	Boichard et al. 2003
19	Beef	4.00-5.40	Backfat	5.352-16.044	*BM6000-BMS745*	5.352-16.044	4.45-8.97	Bovine QTL viewer	Li et al. 2004
			Pre-Weaning average daily gain	10.7	*BM6000-BMS745*	5.352-16.044	4.45-8.97	Iowa State University	Kneeland et al. 2004
			Yield grade	5.352-39.584	*BM6000-BMS1920*	5.352-39.583	4.45-22.25	Bovine QTL viewer	Casas et al. 2003
			Retail product yield	5	*DIK1004-BMS745*	0-15	5.49-8.97	Iowa State University	Casas et al. 2003
19	Beef	24.00-26.00	Backfat	41.56-45.922	*DIK4306-BP20*	41.56-45.922	23.46-28.56	Bovine QTL viewer	Li et al. 2004
			Ribeye area	44	*DIK4058-BMS2503*	43.814-50	24.76-31.11	Iowa State University	Taylor et al. 1998
19	Beef	60-61	Backfat	95.03-98.8	*DIK5199-BMS601*	95.03-98.8	57.80-59.04	Bovine QTL viewer	Li et al. 2004
29	Dairy	11.77-15.15	Protein yield	10	*ARO26-BMC8012*	21.1	13.16	Australian database	Ashwell et al. 2004
			Protein%	20	*BMC8012*	21.1	13.16	Australian database	Mosig et al. 2001
29	Dairy	26.42-27.47	PTA type	37.9	*MNB-150-BMC3224*	29.20-46.67	20.04-32.04	Iowa State University	Ashwell et al. 2005

Supplementary Table 4

			Structurally soundness (legs, feet, penis and prepuce)	37.9	*MNB-150-BMC3224*	29.203-46.67	20.04-32.04	Iowa State University	Ashwell et al. 2005
			Teat Placement	37.9	*MNB-150-BMC3224*	29.203-46.67	20.04-32.04	Iowa State University	Ashwell et al. 2005
			Udder attachment and udder composite index	37.9	*MNB-150-BMC3224*	29.203-46.67	20.04-32.04	Iowa State University	Ashwell et al. 2005
29	Dairy	33.00-34.00	Foot angle	54.5	*BMC3224-BMC6004*	46.673-62.25	32.04-38.89	Iowa State University	Ashwell et al. 2005
			Structurally soundness (legs, feet, penis and prepuce)	54.5	*BMC3224-BMC6004*	46.673-62.25	32.04-38.89	Iowa State University	Ashwell et al. 2005
			Milk yield	28.9	*BMC8012-BMC1206*	21.116-62.53	13.16-38.89	Iowa State University	Viitala et al. 2003
			Protein yield	24.2	*BMC8012-BMC1206*	21.116-62.53	13.16-38.89	Iowa State University	Viitala et al. 2003
29	Beef	7.5-8.50	Marbling Score	6.8-24.48	*ILSTS057-RM044*	6.8-24.48	6.16-18.29	Bovine QTL viewer	MacNeil & Grosz 2002
		18.75-19.45	Hot carcass weight	2.92-41.57	*ILSTS015-HH22*	2.92-41.57	6.79-28.77	Bovine QTL viewer	Kim et al. 2003
29	Beef	27.75-28.68	Marbling Score	40.16-62.53	*RM040-BMC1206*	40.16-62.53	28.22-38.89	Bovine QTL viewer	MacNeil & Grosz 2002
			Meat tenderness	50.40-62.25	*BL1100-BMC6004*	50.40-62.25	34.22-38.89	Bovine QTL viewer	Casas et al. 2003
			Retail product yield	50.40-59.60	*BL1100-MNB-167*	50.40-59.60	34.22-38.22	Bovine QTL viewer	Casas et al. 2003
			Hot carcass weight	50.4-62.25	*BL1100-BMC6004*	50.4-62.25	34.22-38.89	Bovine QTL viewer	Casas et al. 2003
			Body weight at castration	52	*BL1100-MNB-167*	50.40-59.60	34.22-38.22	Iowa State University	Casas et al. 2004

Supplementary Table 4

EHH Results

Chromosome	Dairy/Beef	Regions showing evidence of selection (Mb)	Trait	QTL peak (cM)	Markers	Position (cM)	Position(Mbp,Btau_3.1)	Database	Reference
19	Dairy	62.017-62.184	Milk fat	77.68-86.01	DIK5208-IDVGA-44	77.68-86.01	52.26-55.88	Bovine QTL viewer	Bennewitz *et al.* 2003
19	Dairy	44.417-44.514	Milk yield	69.83	CSSM065	69.83	43.12	Bovine QTL viewer	Shariflou *et al.* 2000
19	Dairy	61.308-61.355	Milk fat	77.68-86.01	DIK5208-IDVGA-44	77.68-86.01	52.26-55.88	Bovine QTL viewer	Bennewitz *et al.* 2003
19	Beef	40.444-40.889	Adjusted fat	62.56-63.18	MB020-GFAP	62.56-63.18	40.26-43.19	Bovine QTL viewer	Kim *et al.* 2003
29	Dairy	11.655-11.739	Temperament	20	BMS764-BMC8012	11.293-21.11	7.98-13.16	Iowa State University	Hiendleder *et al.* 2003
			Milking speed and temperament	2.923-21.116	ILSTS015-BMC8012	2.923-21.116	1.44-13.16	Bovine QTL viewer	Hiendleder *et al.* 2003
			Protein	16.7	MNB-131-BMC8012	11.593-21.11	8.43-13.16	Iowa State University	Ashwell *et al.* 2004
29	Dairy	29.840-31.096	Structurally soundness (lega, feet, penis and prepuce), Teat Placement, Udder attachment and udder composite index	37.9	MNB-150-BMC3224	29.203-46.67	20.04-32.04	Iowa State University	Ashwell *et al.* 2005
			Protein	24.2	BMC8012-BMC1206	21.116-62.53	13.16-38.89	Iowa State University	Viitala *et al.* 2003
			Milk	28.9	BMC8012-BMC1206	21.116-62.53	13.16-38.89	Iowa State University	Viitala *et al.* 2003

29	Dairy	31.807-32.078	Structurally soundness (lega, feet, penis and prepuce), Teat Placement, Udder attachment and udder composite index	37.9	MNB-150-BMC3224	29.203-46.67	20.04-32.04	Iowa State University	Ashwell *et al.* 2005
			Protein	24.2	BMC8012-BMC1206	21.116-62.53	13.16-38.89	Iowa State University	Viitala *et al.* 2003
			Milk	28.9	BMC8012-BMC1206	21.116-62.53	13.16-38.89	Iowa State University	Viitala *et al.* 2003
29	Dairy	33.693-34.136	Protein	24.2	BMC8012-BMC1206	21.116-62.53	13.16-38.89	Iowa State University	Viitala *et al.* 2003
			Milk	28.9	BMC8012-BMC1206	21.116-62.53	13.16-38.89	Iowa State University	Viitala *et al.* 2003
			Structurally soundness (legs, feet, penis and prepuce), Teat Placement, Udder attachment and udder composite index	37.9	MNB-150-BMC3224	29.203-46.67	20.04-32.04	Iowa State University	Ashwell *et al.* 2005
29	Beef	7.767-8.006	Marbling Score	6.8-24.48	ILSTS057-RM044	6.8-24.48	6.16-18.29	Bovine QTL viewer	MacNeil and Grosz 2002
			Hot carcass weight	2.92-41.57	ILSTS015-HH22	2.92-41.57	6.79-28.77	Bovine QTL viewer	Kim *et al.* 2003

References

Ashwell M.S., Heyen D.W., Sonstegard T.S., Van Tassell C.P., Da Y., VanRaden P.M., Ron M., Weller J.I. & Lewin H.A. (2004) Detection of quantitative trait loci affecting milk production, health and reproductive traits in holstein cattle. *Journal of Dairy Science* **87,** 468-75.

Ashwell M.S., Heyen D.W., Weller J.I., Ron M., Sonstegard T.S., Van Tassell C.P. & Lewin H.A. (2005) Detection of quantitative trait loci influencing conformation traits and calving ease in Holstein-Friesian cattle. *Journal of Dairy Science* **88**, 4111-9.

Supplementary Table 4

Bennewitz J., Reinsch N., Grohs C., Leveziel H., Malafosse A., Thomsen H., Xu N., Looft C., Kuhn C., Brockmann G.A., Schwerin M., Weimann C., Hiendleder S., Erhardt G., Medjugorac I., Russ I., Forster M., Brenig B., Reinhardt F., Reents R., Averdunk G., Blumel J., Boichard D. & Kalm E. (2003) Combined analysis of data from two granddaughter designs: A simple strategy for QTL confirmation and increasing experimental power in dairy cattle. *Genetics Selection Evolution* **35**, 319-38.

Boichard D., Grohs C., Bourgeois F., Cerqueira F., Faugeras R., Neau A., Rupp R., Amigues Y., Boscher M.Y. & Leveziel H. (2003) Detection of genes influencing economic traits in three French dairy cattle breeds. *Genetics Selection Evolution* **35**, 77-101.

Casas E., Shackelford S.D., Keele J.W., Koohmaraie M., Smith T. P. & Stone R.T. (2003) Detection of quantitative trait loci for growth and carcass composition in cattle. *Journal of Animal Science* **81**, 2976-83.

Casas E., Lunstra D.D. & Stone R.T. (2004) Quantitative trait loci for male reproductive traits in beef cattle. *Animal Genetics* **35**, 451-3.

Hiendleder S., Thomsen H., Reinsch N., Bennewitz J., Leyhe-Horn B., Loft C., Xu N., Medjugorac I., Russ I., Kuhn C., Brockmann G.A., Blumel J., Brenig B., Reinhardt F., Reents R., Averdunk G., Schwerin M., Forster M., Kalm E. & Erhardt G. (2003) Mapping of QTL for body conformation and behaviour in cattle. *Journal of Heredity* **94**, 496-506.

Kim J.J., Farnir F., Savell J. & Taylor J.F. (2003) Detection of quantitative trait loci for growth and beef carcass fatness traits in a cross between *Bos taurus* (Angus) and *Bos indicus* (Brahman) cattle. *Journal of Animal Science* **81**, 1933-42.

Kneeland J., Li C., Basarab J., Snelling W.M., Benkel B., Murdoch B., Hansen C. & Moore S.S. (2004) Identification and fine mapping of quantitative trait loci for growth traits on bovine chromosomes 2, 6, 14, 19, 21 and 23 within one commercial line of *Bos taurus*. *Journal of Animal Science* **82**, 3405-14

Li C., Basarab J., Snelling W.M., Benkel B., Kneeland J., Murdoch B., Hansen C. & Moore S.S. (2004) Identification and fine mapping of quantitative trait loci for backfat on bovine chromosomes 2, 5, 6, 19, 21 and 23 in a commercial line of *Bos taurus*. *Journal of Animal Science* **82**, 967-72.

MacNeil M.D. & Grosz M.D. (2002) Genome-wide scans for QTL affecting carcass traits in Hereford × composite double backcross populations. *Journal of Animal Science* **80**, 2316-24.

Mosig M.O., Lipkin E., Khutoreskaya G., Tchourzyna E., Soller M. & Friedmann A. (2001) A whole-genome scan for quantitative trait loci affecting milk protein percentage in Israeli-Holstein cattle, by means of selective milk DNA pooling in a daughter design, using an adjusted false discovery rate criterion. *Genetics* **157**, 1683-98.

Shariflou M.R., Moran C. & Nicholas F.W. (2000) Association of the Leu127 variant of the bovine *growth hormone* (bGH) gene with increased yield of milk, fat and protein in Australian Holstein-Friesians. *Australian Journal of Agricultural Research* **51**, 515-22.

Taylor J.F., Coutinho L.L., Herring K.L., Gallagher D.S.Jr, Brenneman R.A., Burney N., Sanders J.O., Turner J.W., Smith S.B., Miller R.K., Savell J.W. & Davis S.K. (1998) Candidate gene analysis of *GH1* for effects on growth and carcass composition of cattle. *Animal Genetics* **29,** 194-201.

Viitala S.M., Schulman N.F., de Koning D.J., Elo K., Kinos R., Virta A., Virta J., Maki-Tanila A. & Vilkki J.H. (2003) Quantitative trait loci affecting milk production traits in Finnish Ayrshire dairy cattle. *Journal of Dairy Science* **86**, 1828-36.
