# Supplementary figures and images for: Linkage disequilibrium and signatures of selection on chromosomes 19 and 29 in beef and dairy cattle
Source: Anim Genet. 2008 Dec;39(6):597–605. doi: 10.1111/j.1365-2052.2008.01772.x (PMC2659388; doi:10.1111/j.1365-2052.2008.01772.x)

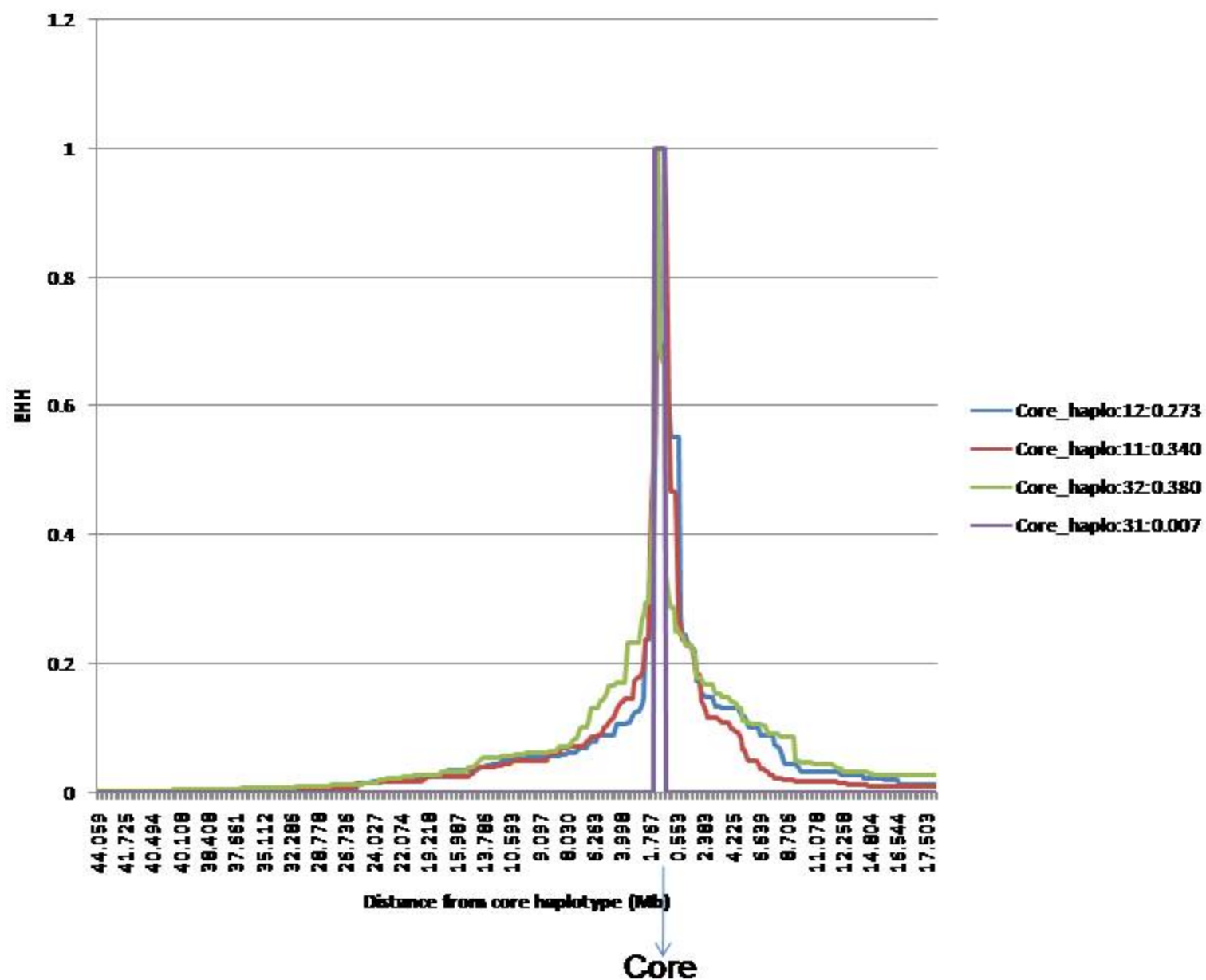

**Supplementary Figure 1**

Supplement: Supplementary file 5 [file age0039-0597-SD5.pdf]

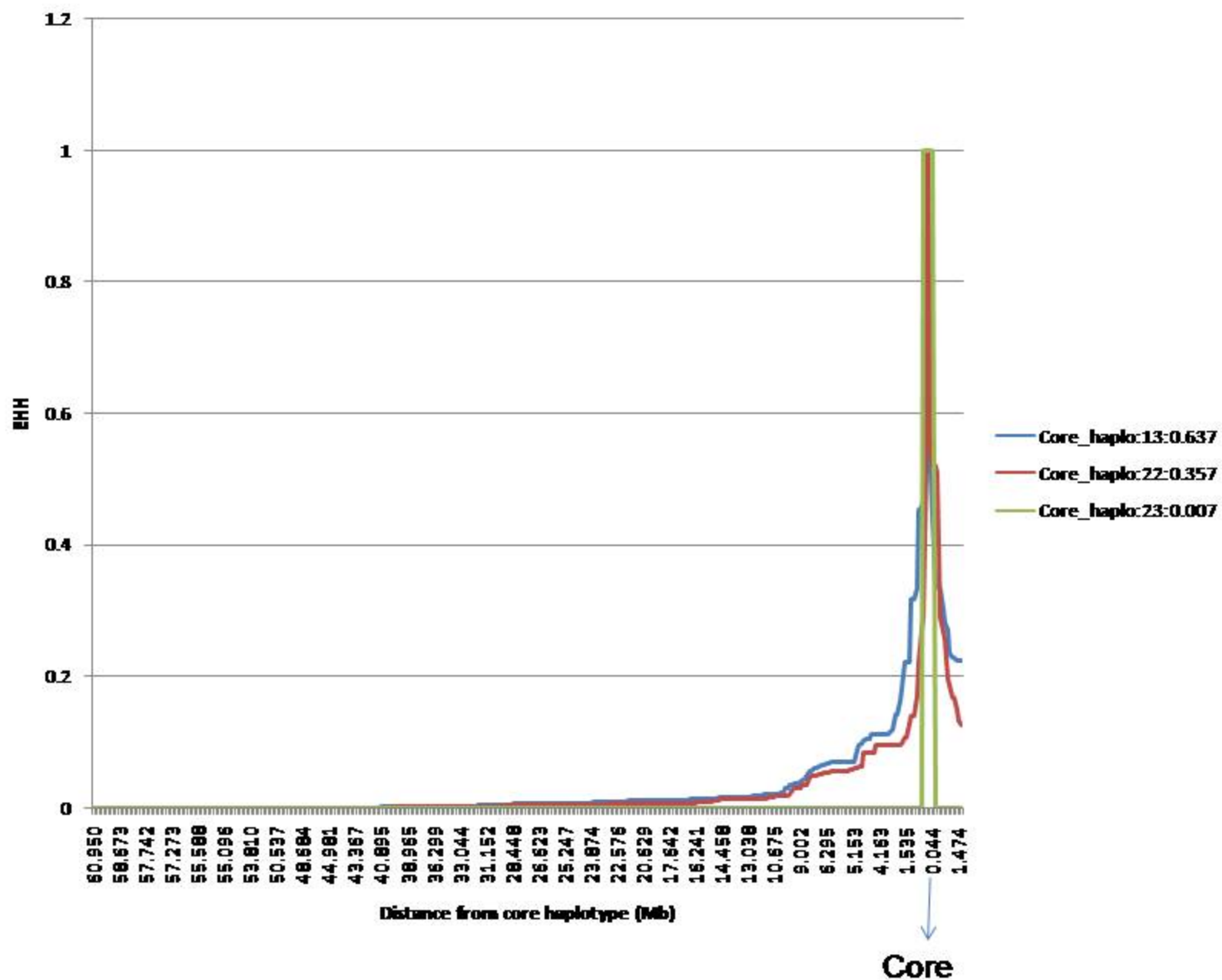

**Supplementary Figure 2**

Supplement: Supplementary file 6 [file age0039-0597-SD6.pdf]

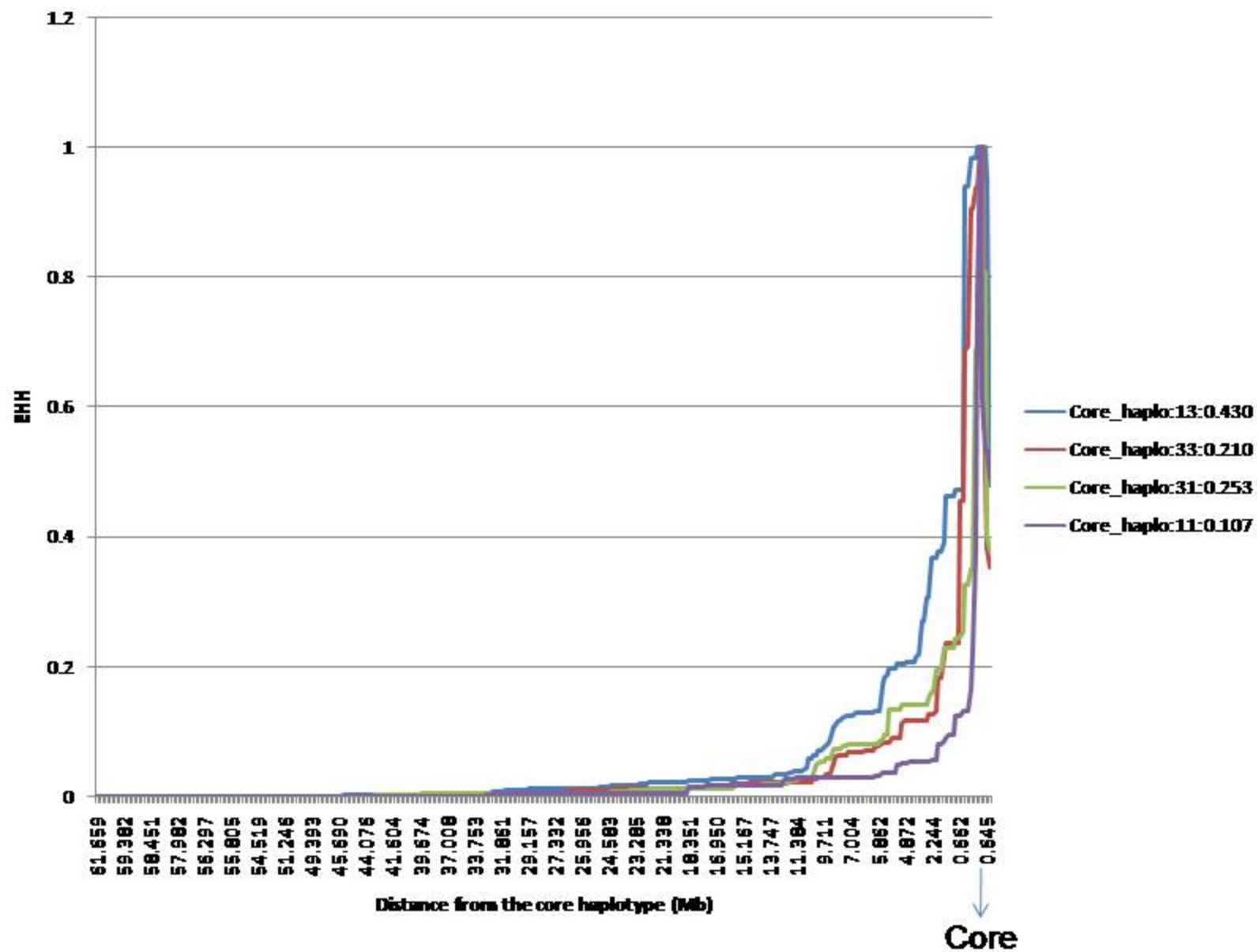

**Supplementary Figure 3**

Supplement: Supplementary file 7 [file age0039-0597-SD7.pdf]

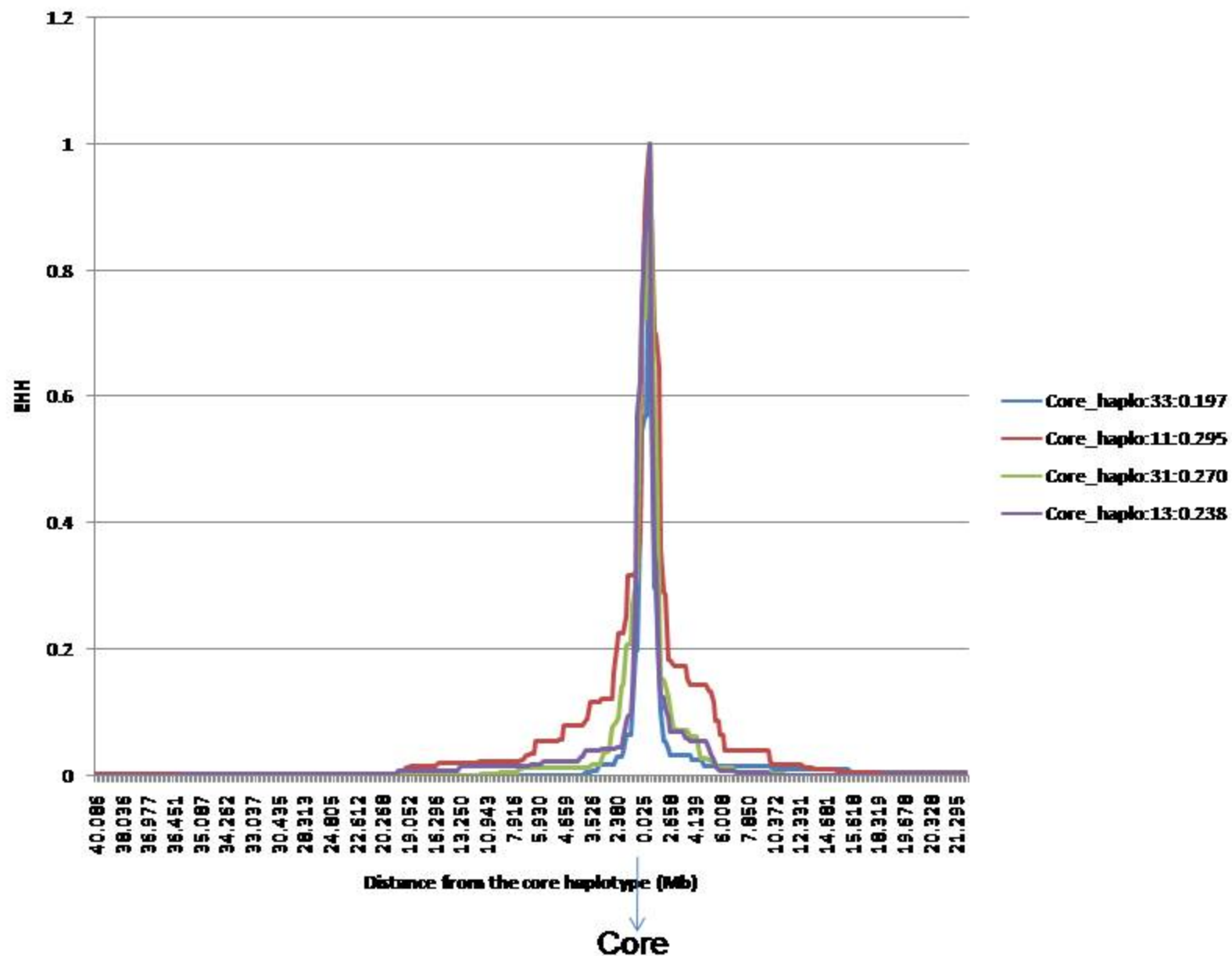

**Supplementary Figure 4**

Supplement: Supplementary file 8 [file age0039-0597-SD8.pdf]

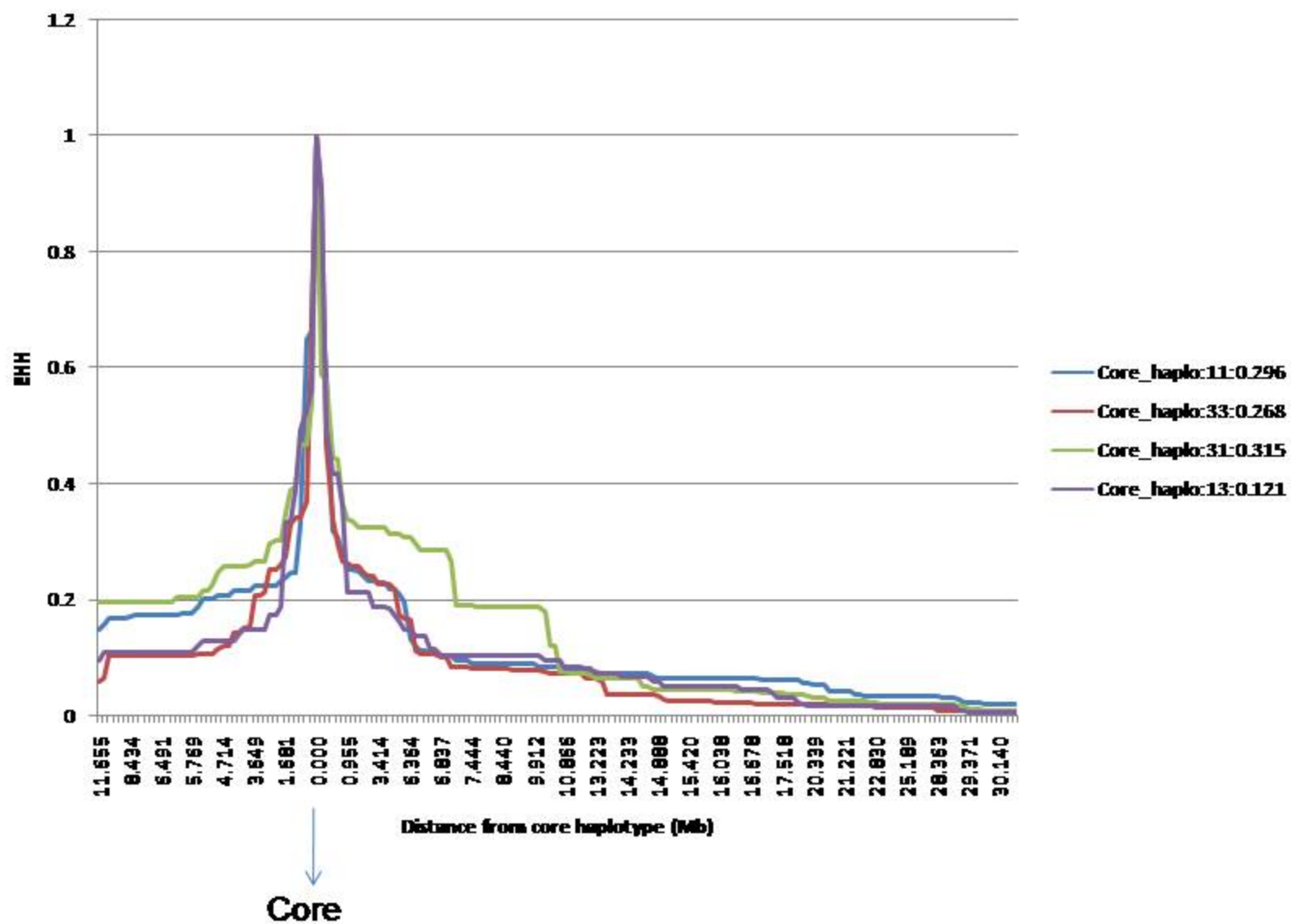

**Supplementary Figure 5**

Supplement: Supplementary file 9 [file age0039-0597-SD9.pdf]

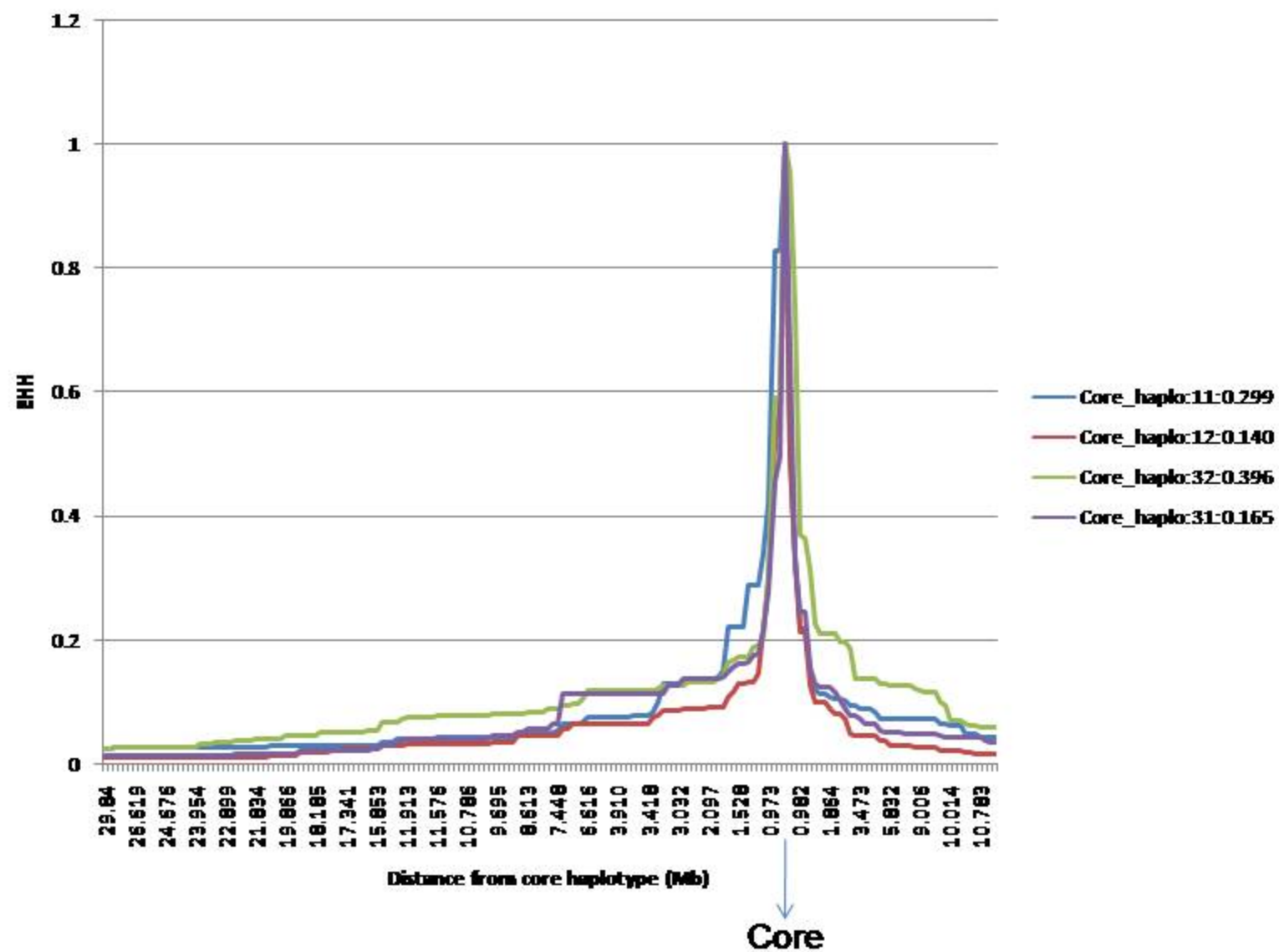

**Supplementary Figure 6**

Supplement: Supplementary file 10 [file age0039-0597-SD10.pdf]

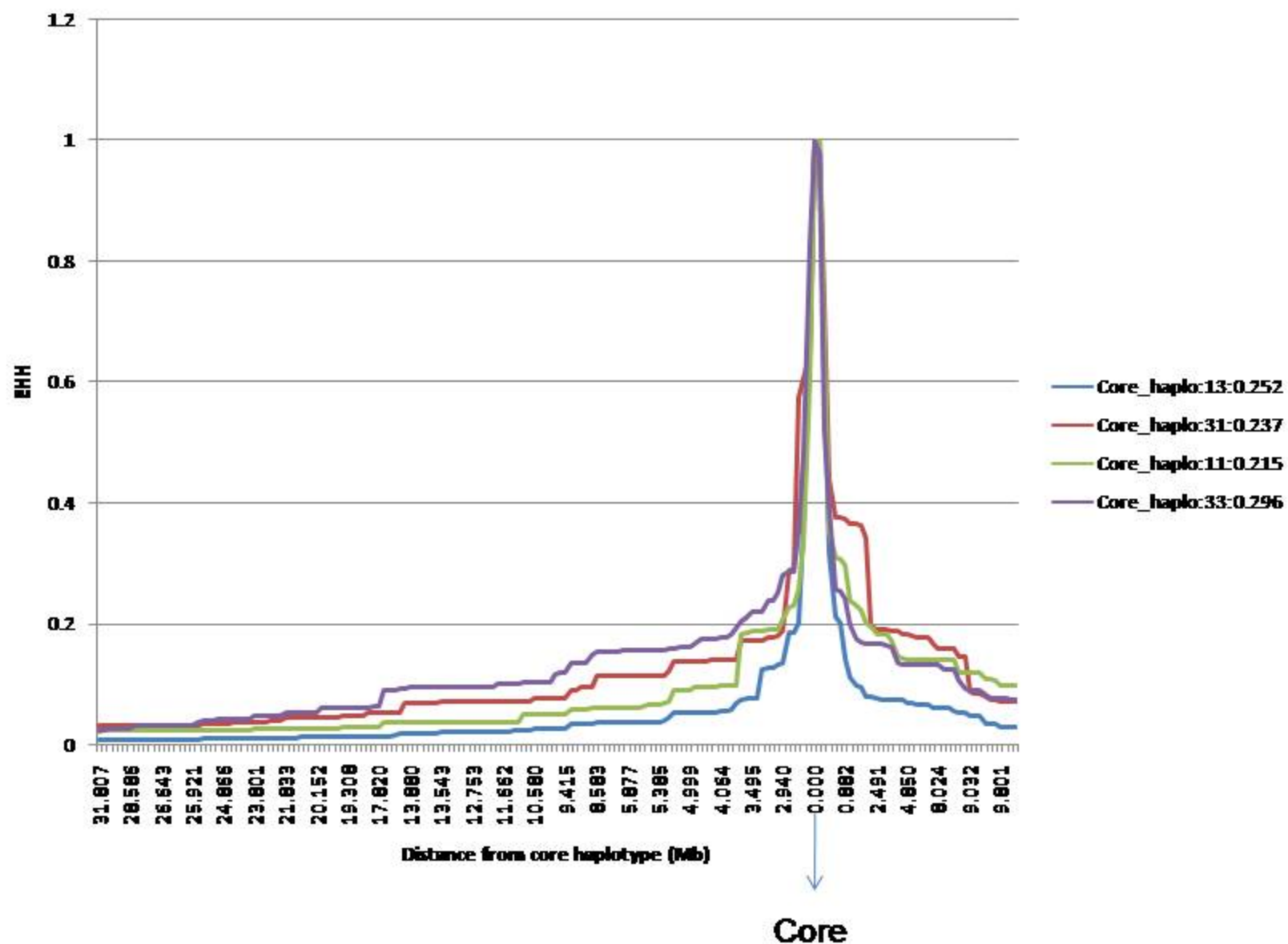

**Supplementary Figure 7**

Supplement: Supplementary file 11 [file age0039-0597-SD11.pdf]

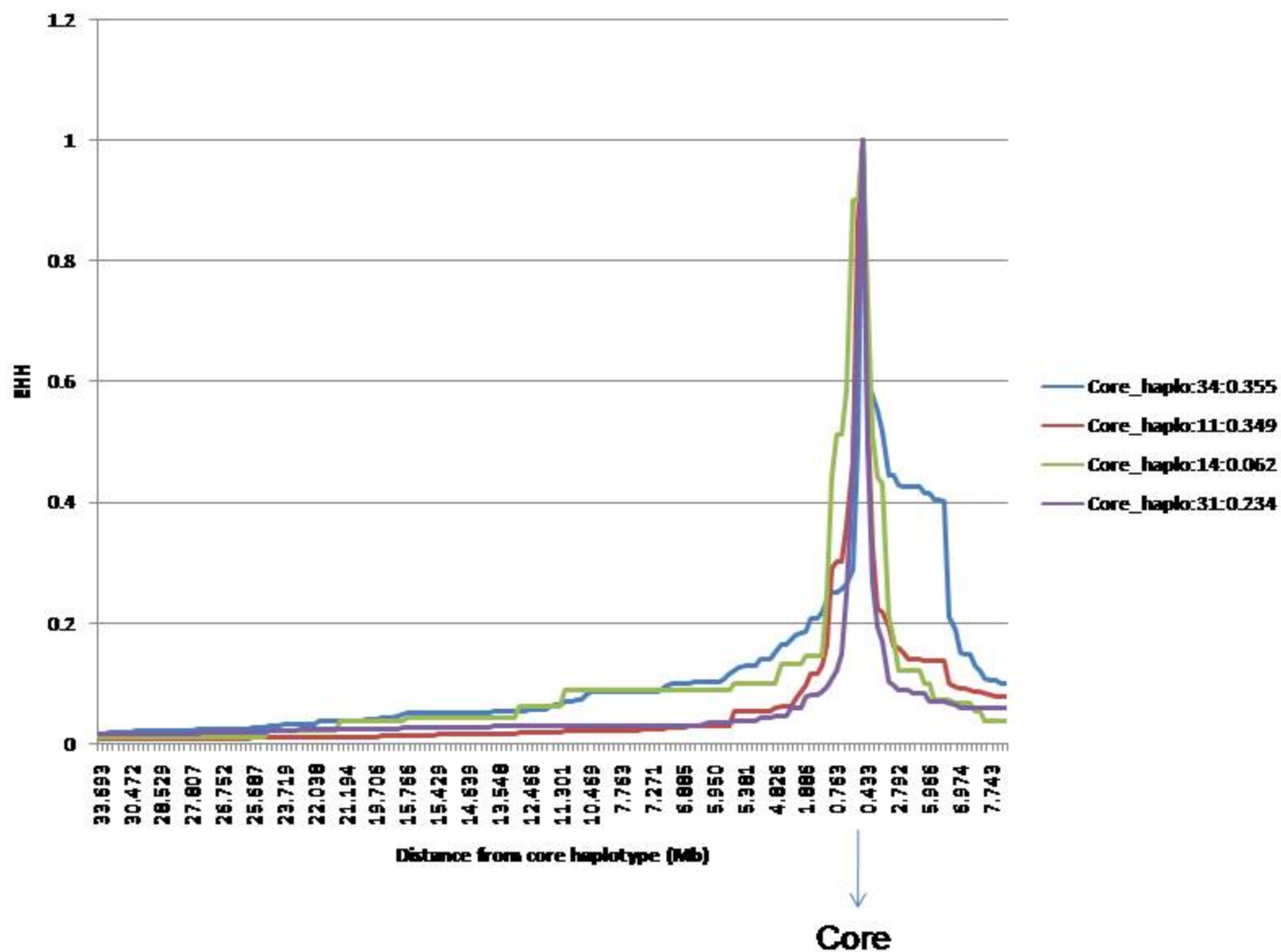

**Supplementary Figure 8**

Supplement: Supplementary file 12 [file age0039-0597-SD12.pdf]

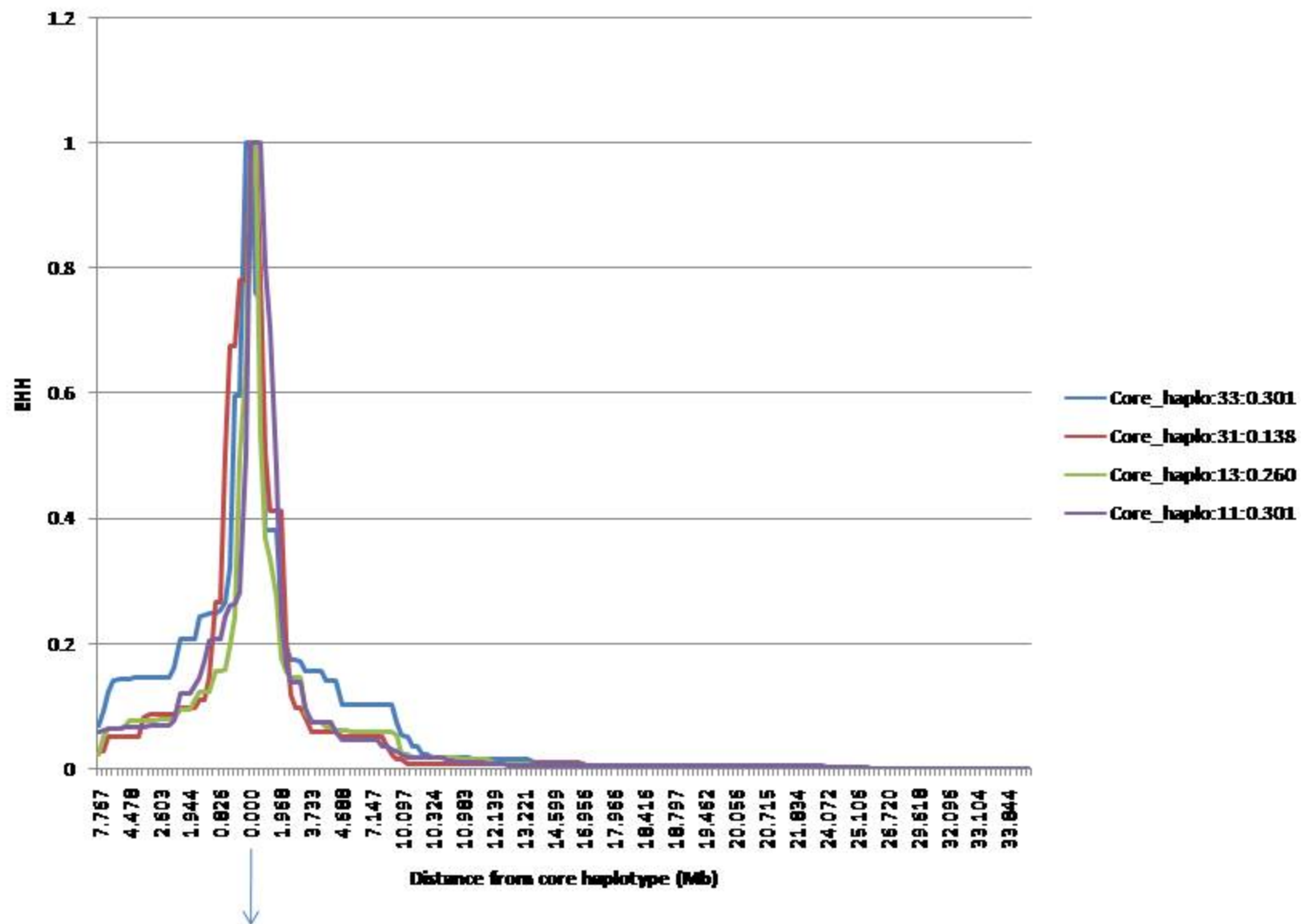

Core

**Supplementary Figure 9**

Supplement: Supplementary file 13 [file age0039-0597-SD13.pdf]

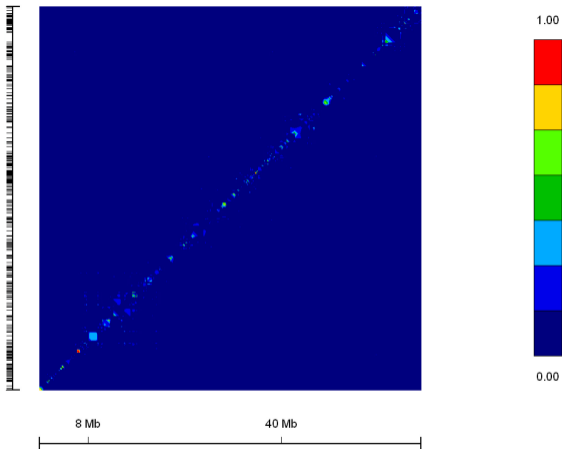

Supplementary Figure 10

Supplement: Supplementary file 14 [file age0039-0597-SD14.pdf]

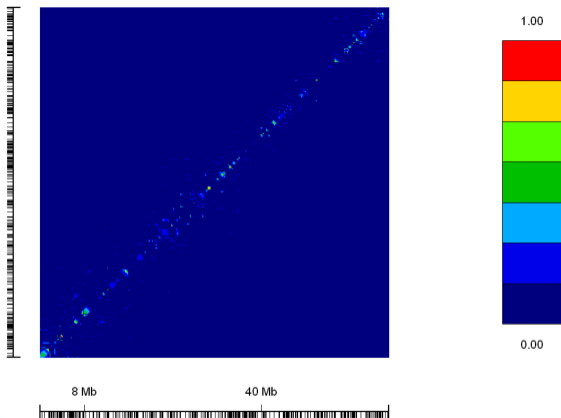

Supplementary Figure 11

Supplement: Supplementary file 15 [file age0039-0597-SD15.pdf]

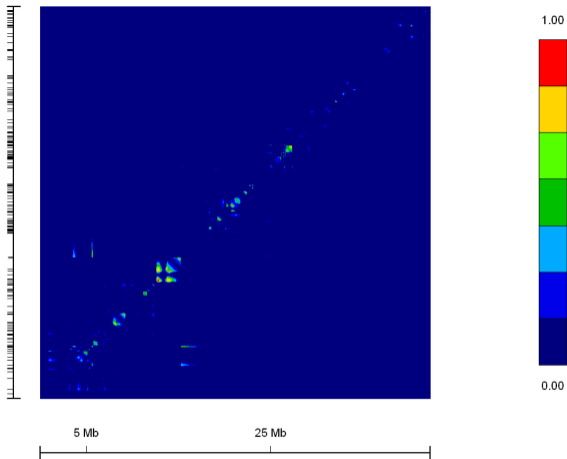

Supplementary Figure 12

Supplement: Supplementary file 16 [file age0039-0597-SD16.pdf]

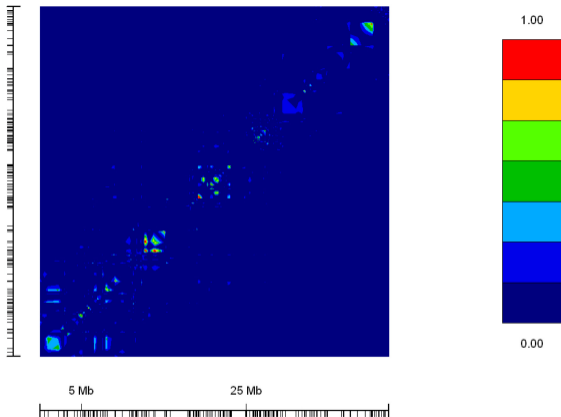

Supplementary Figure 13

Supplement: Supplementary file 17 [file age0039-0597-SD17.pdf]
